# Bacterial genome editing by coupling Cre-*lox* and CRISPR-Cas9 systems

**DOI:** 10.1371/journal.pone.0241867

**Published:** 2020-11-04

**Authors:** Hualan Liu, David S. Robinson, Zong-Yen Wu, Rita Kuo, Yasuo Yoshikuni, Ian K. Blaby, Jan-Fang Cheng

**Affiliations:** 1 US Department of Energy Joint Genome Institute, Berkeley, California, United States of America; 2 Environmental Genomics and Systems Biology Division, Lawrence Berkeley National Laboratory, Berkeley, California, United States of America; 3 Department of Veterinary Medicine, National Chung Hsing University, Taichung, Taiwan, ROC; 4 Biological Systems and Engineering Division, Lawrence Berkeley National Laboratory, Berkeley, California, United States of America; University of Illinois at Urbana-Champaign, UNITED STATES

## Abstract

The past decade has been a golden age for microbiology, marked by the discovery of an unprecedented increase in the number of novel bacterial species. Yet gaining biological knowledge of those organisms has not kept pace with sequencing efforts. To unlock this genetic potential there is an urgent need for generic (i.e. non-species specific) genetic toolboxes. Recently, we developed a method, termed chassis-independent recombinase-assisted genome engineering (CRAGE), enabling the integration and expression of large complex gene clusters directly into the chromosomes of diverse bacteria. Here we expand upon this technology by incorporating CRISPR-Cas9 allowing precise genome editing across multiple bacterial species. To do that we have developed a landing pad that carries one wild-type and two mutant *lox* sites to allow integration of foreign DNA at two locations through Cre-*lox* recombinase-mediated cassette exchange (RMCE). The first RMCE event is to integrate the *Cas9* and the DNA repair protein genes *RecET*, and the second RMCE event enables the integration of customized sgRNA and a repair template. Following this workflow, we achieved precise genome editing in four different gammaproteobacterial species. We also show that the inserted landing pad and the entire editing machinery can be removed scarlessly after editing. We report here the construction of a single landing pad transposon and demonstrate its functionality across multiple species. The modular design of the landing pad and accessory vectors allows design and assembly of genome editing platforms for other organisms in a similar way. We believe this approach will greatly expand the list of bacteria amenable to genetic manipulation and provides the means to advance our understanding of the microbial world.

## Introduction

Second and third generation sequencing technologies, and in particular, shotgun metagenomics, have expanded our knowledge of the microbial diversity dramatically [1]. The development of new culturing approaches and the isolation of microbes from wide-ranging environmental sources has drawn the attention of both the medical and industrial fields, with particular interests in those organisms exhibiting unique biophysical and/or biochemical properties [2–5]. However, our ability to understand and harness this novel functional diversity is hindered by limited genetic tools [6–8].

To help address the limitation of universally applicable molecular approaches, we previously developed the CRAGE technology [9]. CRAGE comprises a two-plasmid system consisting of a 2-*lox* landing pad vector and an accessory vector. The landing pad vector contains the mariner transposase on the backbone, and in between the two inverted repeats (IR) is the transposon. The transposon has two *lox* sites, with a selectable marker and Cre recombinase in between. The accessory vector contains the same two *lox* sites, whose orientation matches those of the landing pad vector, flanking the desired non-native genes/pathway. The landing pad was introduced into the chromosome via mariner-based transposon insertion, and the custom pathway integrated into the landing via Cre-*lox* recombinase-mediated cassette exchange (RMCE). Since both the transposon and the Cre-*lox* recombineering systems were applied to diverse hosts, CRAGE enabled insertion of large biosynthetic gene clusters into 25 diverse gammaproteobacterial species. However, this approach does not provide capabilities beyond insertion, such as gene deletions or modulated gene expression.

The type II CRISPR-Cas system from *Streptococcus pyogenes*, incorporating the signature endonuclease Cas9, has rapidly become a widely adopted tool for genome editing due to its near universal application and ease of target programming [10–12]. It is able to carry out complex genome editing tasks, including gene deletions, SNPs, and gene insertions. With further modification on the catalytic activity of Cas9, other genetic perturbations are possible. These include using a catalytically inactive version of Cas9 (dCas9) for transcriptional repression (CRISPRi) [13] and also transcriptional activation (CRISPRa) could be achieved by fusing a transcriptional activator to the C-terminal of dCas9 [14, 15].

The first bacterial CRISPR-Cas9 genome editing was demonstrated in *Streptococcus pneumoniae* and *Escherichia coli* in 2013 [16]. While the number of bacteria documented to have been engineered by CRISPR-Cas9 editing is expanding, the pace for developing such tools is slow [17], and several challenges have been associated with this technique. First, high level expression of *Cas9* could be toxic in some species, which may be exacerbated when expressed from a high copy plasmid [18, 19]. Second, many bacteria lack strong DNA repair systems to fix the double strand breaks during editing, which could cause cell death [16, 20]. Finally, many currently described CRISPR-Cas9 systems for bacterial editing employ one or several plasmids, and thus an efficient plasmid curing strategy is preferred for sequential editing [21, 22]. Given these obstacles, developing CRISPR-Cas9 functionality for a new bacterial isolate can be a laborious process, and does not match the pace of identification of new microbial species [5]. Versatile approaches with broad applicability to many species would enable rapid genetic interrogation of many organisms simultaneously without the need to develop custom methodologies [23].

In this work, we aimed to develop a method that combines the universality of both CRISPR-Cas9 and CRAGE, enabling the breadth of CRISPR-mediated editing to be applied as widely as Cre-*lox* functionality. Taking advantage of the broad host functionality of those genetic parts, this single genetic engineering system enables us to genetically manipulate novel bacterial isolates across species, and thus accelere the rate at which they can be investigated.

Towards this goal, we first integrated a Cre-*lox* system into the chromosomes of target species. Cas9 machinery was introduced by RMCE, then the sgRNA and repair template were introduced via a second round of RMCE. This offers an alternative solution for organisms that do not have a replicative plasmid system. To demonstrate our approach, we used this system to carry out targeted editing in four taxonomically diverse gammaproteobacterial species [24]. We systematically evaluated the editing efficiency across 10 different loci in *Photorhabdus luminescens*, where the editing efficiency varied from 31% to 100%. We demonstrated that the Cas9 machinery could be removed scarlessly from *P*. *luminescens* subsequent to achieving the desired edits. Since the key components (transposase, Cre-*lox*, CRISPR-Cas9) for this system are known to be functional across different phyla [17, 25–28], we anticipate they could be used as standard genetic parts with relatively minor modification to provide applications to a broad range of organisms.

## Results

### Designing a genome editing approach using the combination of a 3-*lox* landing pad and CRISPR-Cas9

**Fig 1:**
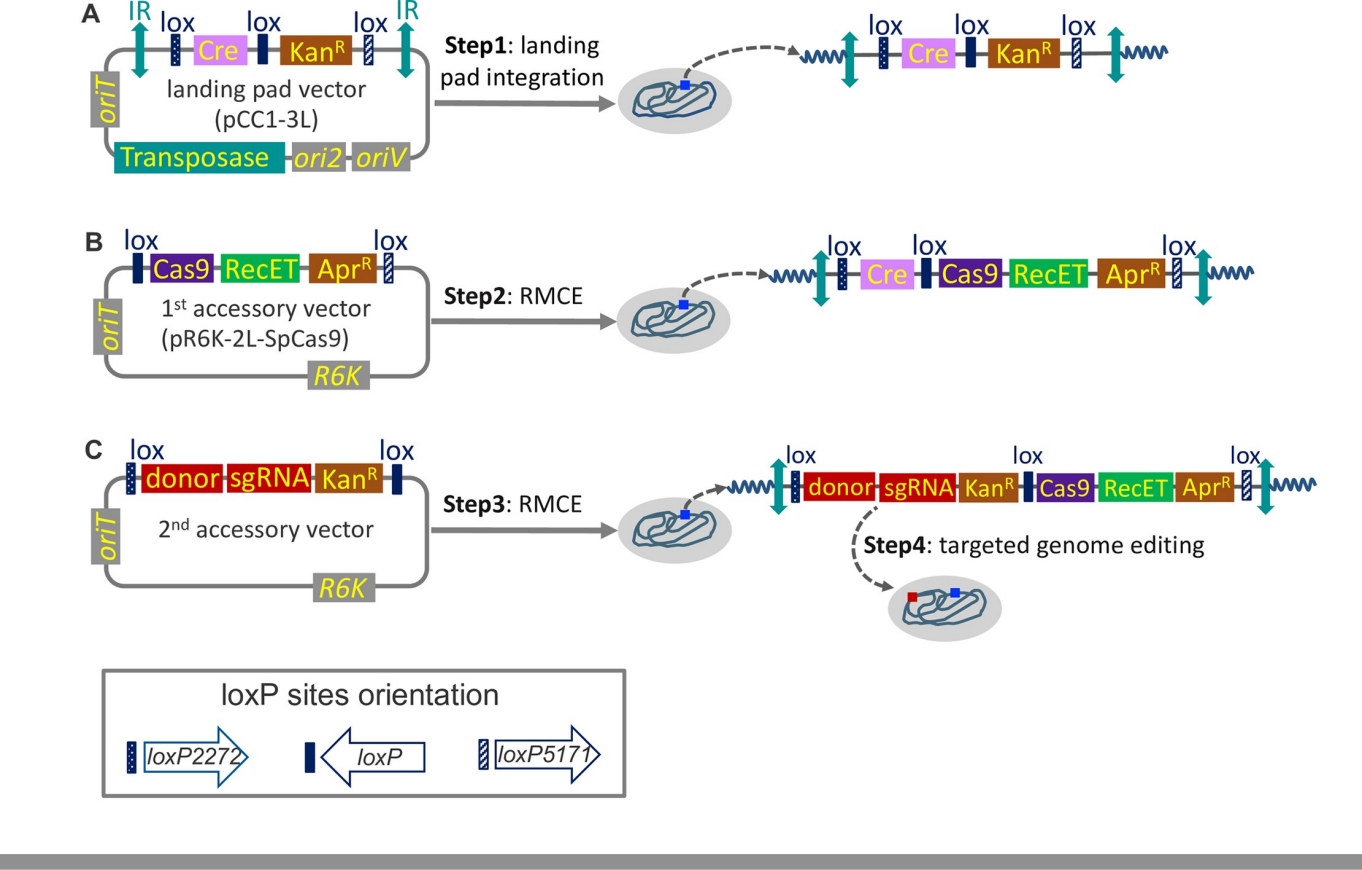
Overview of steps involved in the cross-species CRISPR-Cas9 genome editing strategy (not drawn to scale). (A): Chromosomal integration of the landing pad vector by mariner-based transposon system. (B): Chromosome integration of the *Cas9* and *RecET* via RMCE. (C): Chromosome integration of the sgRNA and donor template via RMCE, followed by genome editing at the specific target site. The orientation of *lox* sites was kept consistent in all the vectors. *IR* is the transposon-specific inverted repeats. *oriT* is the origin of transfer. *KanR* and *AprR* are antibiotic resistance cassettes for kanamycin and apramycin, respectively. *OriS*, *oriV* and *R6K* are different replication origins.

Our overall approach is illustrated in Fig 1. To achieve stable expression of the components for CRISPR-Cas9 genome editing, we designed a 3-*lox* CRAGE based approach. This design allows editing machinery to be inserted into the 1^st^ location (between wild type *lox*P and *lox*5171), and target-specific sgRNA and DNA repair donor template to be inserted into the 2^nd^ location (between *lox*2272 and *lox*P) of the landing pad. The overall workflow utilizes three vectors: a landing pad vector and two accessory vectors. Each was introduced to the host by conjugation, which is a well-established strategy for introducing foreign DNA into diverse organisms [29].

The transposon-based landing pad plasmid used in this study (pCC1-3L) contains a wild-type and two mutated *lox* sites separating Cre and a kanamycin selectable marker gene (Fig 1A). We employed wild-type *lox*P, *lox*2272, and *lox*5171 since these sites can recombine efficiently with themselves, but not or only rarely with each other [30, 31]. The transposase and kanamycin resistant gene and their promoters were from the original pKMW2 transposon because it has been shown to be functional in at least 22 different Proteobacteria from the classes of alpha, beta and gamma [25]. We used the *Cas9* promoter originating from *S*. *pyogenes* to drive the expression of Cre. Each of these components were cloned into a single copy vector pCC1FOS (Epicentre, Madison, USA) (to avoid intermolecular recombination that would occur in a non-single copy vector) to generate pCC1-3L.

Once pCC1-3L was conjugated into the recipient strain, expression of the transposase catalyzes integration of the transposon into the genome (Fig 1A). To minimize the likelihood of gene disruption of the strain, transformants containing intergenic insertions of the landing pad were selected whenever possible for further modification and editing (see methods section).

Subsequent to the integration of the landing pad, the sequential introduction of two accessory vectors is necessary to deliver Cas9 and the editing-specific sgRNA and repair template. The first accessory vector, pR6K-loxWT5171-SpCas9 (Fig 1B), contains *lox* sites matching those flanking the kanamycin selectable marker in the landing pad (S1 Fig). Between this pair of *lox* sites are the original *S*. *pyogenes Cas9* driven by its native promoter, the bacteriophage RecET recombination system under the arabinose inducible promoter *ParaB*, and an apramycin selectable marker. We incorporated the *rac* prophage derived recombination system RecE and RecT to account for the possibility that the host-encoded homologous recombination is not efficient enough to repair the double-stranded break generated by Cas9.

The first accessory vector carries the R6K origin of replication. The replication of R6K derived plasmids is dependent on the *pir* gene-encoded π protein for replication. Consequently, when transformed into a host lacking the *pir* gene, and selected with apramycin, only transformants that went through Cre-assisted cassette exchange will grow (Fig 1B), thus providing selection for the first RMCE.

The second accessory vector also carries the R6K replication origin and a pair of *lox* sites flanking the sgRNA, the donor template for DNA repair and kanamycin marker (recycled after the first RMCE) (S1 Fig). This second RMCE event resulted in the loss of Cre and editing at the target site. (Fig 1C). Our decision to integrate the sgRNA and the repair template into the chromosome came from the concern that a relatively high expression level of the gRNA is necessary for efficient editing [32]. This way both the gRNA and the repair template is propagating with the cells, so that even if there is a delay of gRNA expression the repair template would always be present to fix the double-stranded breaks.

### Genome editing in four gammaproteobacterial species

#### Chromosome integration of 3-*lox* landing pads

To evaluate the broad application of our approach, we tested our methods in four different gammaproteobacteria: *Aeromonas salmonicida subsp*. *pectinolytica* 34mel, *Pseudomonas simiae* WCS417, *Shewanella oneidensis* MR-1, and *Photorhabdus luminescens subsp*. *laumondii* TT01. The landing pad vector was successfully introduced into all four strains by conjugation and kanamycin-resistant transformants were obtained. The landing pad integration sites in those transformants were identified using inverse PCR and sequencing [33]. We then selected a transformant with the transposon insertion site that has the least impact on cell growth (in comparison to a wild-type strain) as determined by a growth curve assay (S2 Fig). The 3-*lox* landing pad integration sites for each selected strain used for downstream genome editing are listed in S2 Table.

#### Integration of *Cas9* and recombinase genes

We introduced the first accessory vector, pR6K-loxWT5171-SpCas9, into each gammaproteobacterial parent strain again by conjugation. RMCE-positive colonies were first selected for gain of resistance to apramycin and loss of resistance to kanamycin on agar plates. Then we tested the presence of *Cas9* gene and absence of the pR6K vector backbone by PCR amplification of the genomic DNA to confirm successful cassette exchange of the first accessory vector (data not shown).

#### Insertion of sgRNA and donor DNA to achieve genome editing

To rapidly test CRISPR-Cas9 editing in each strain, we identified genes with either a clear visual phenotype that can easily be assayed, or genes involved in secondary metabolites production (Table 1). For *A*. *salmonicida subsp*. *pectinolytica* 34mel, we selected the *hpd* gene, encoding 4-hydroxyphenylpyruvate dioxygenase. Loss of *hpd* abolishes production of melanin, leading to the loss of the dark brown color of the colony [34]. For *P*. *simiae* WCS417, we selected the gene encoding the thioesterase, which is involved in the final step in the biosynthesis of pyoverdine, a siderophore [35, 36]. For *S*. *oneidensis* MR-1, we chose the *flaG* gene as a deletion target. Mutation of *flaG* leads to the disassembly of the flagella, and thus loss of motility. This mutant phenotype can be easily assayed on soft agar plates [37]. For *P*. *luminescens subsp*. *laumondii* TT01, we selected 10 different target genes with each encoding a predicted non-ribosomal peptide/polyketide synthase (Table 1).

**Table 1:** Summary of successful genome editing in four different bacteria species.

Strain ID (landing pad insertion site (strand))	Targeted gene/pathway^a^	gRNA sequence	Editing type (size, chromosome location^b^)
*Aeromonas salmonicida subsp*. *Pectinolytica* 34mel, (3403527–3403528 (-))	*hpd* (locus_tag: Asalp_21790)	AGAACCCAGCGATGCGTAAC	Deletion (1512 bp, 2343500–2345011)
*Pseudomonas simiae* WCS417r, (1774856–1774,857 (-))	*Thioesterase* (locus_tag: PS417_19550)	GCATACAGCGTGTTTCAGTT	Deletion (520 bp, 4252971–4253490)
*Shewanella oneidensis* MR-1, (3069441-3069442(+))	flaG (locus_tag: SO_3236)	ACAAATGCTTATGATGGTGAGGTATAGCTA	Deletion (909 bp, 3375174–3376082)
*Photorhabdus luminescens* subsp. *laumondii* TT01, (4881822–4881823 (+))	NRPS^c^_1 (locus_tag: PluTT01m_04610)	TGCAGATTTATATTAATAGC	Deletion (3294 bp, 1020927–1024220)
	NRPS_2 (locus_tag: PluTT01m_05670)	TAAATAGTTATGATAACTAT	Deletion (2300 bp, 1276716–1279015)
	NRPS_3 (locus_tag: PluTT01m_06165–06180)	GTATACATATCCAAGTTACT	Deletion (7705 bp, 1391134–1398838)
	NRPS_4 (locus_tag: PluTT01m_09665–09670)	ATCGAGATATTCTTATTTAT	Deletion (5117 bp, 2235387–2240503)
	NRPS_5 (locus_tag: PluTT01m_11950–11970)	CCCTATTCCGTTAGCCCTGA	Deletion (8003 bp, 2713290–2721292)
	NRPS_6 (locus_tag: PluTT01m_13700)	TACCCGTTTTTATTTCACCG	Deletion (7011 bp, 3166873–3173883)
	NRPS_7 (locus_tag: PluTT01m_16070)	AGTGCCGTTCCGTCATCTGG	Deletion (6034 bp, 3656505–3662538)
	NRPS_9 (locus_tag: PluTT01m_16105)	CGGTATCTAAGTCTCTTGGA	Deletion (5066 bp, 3683630–3688695)
	NRPS_10 (locus_tag: PluTT01m_16815)	CCAAGAGAATGTGCTGTCTG	Deletion (6067 bp, 3875195–3881261)
	NRPS_11 (locus_tag: PluTT01m_18130–18145)	AAACCTGTATGTATCGGGAG	Deletion (6188 bp, 4162085–4168272)

a: The gRNA targeting site might be in the upstream regulatory region or in the open reading frame.

b: the position of the start and the end of the deletion on the chromosome.

c: NRPS stands for non-ribosomal peptide/polyketide synthase gene cluster.

**Fig 2:**
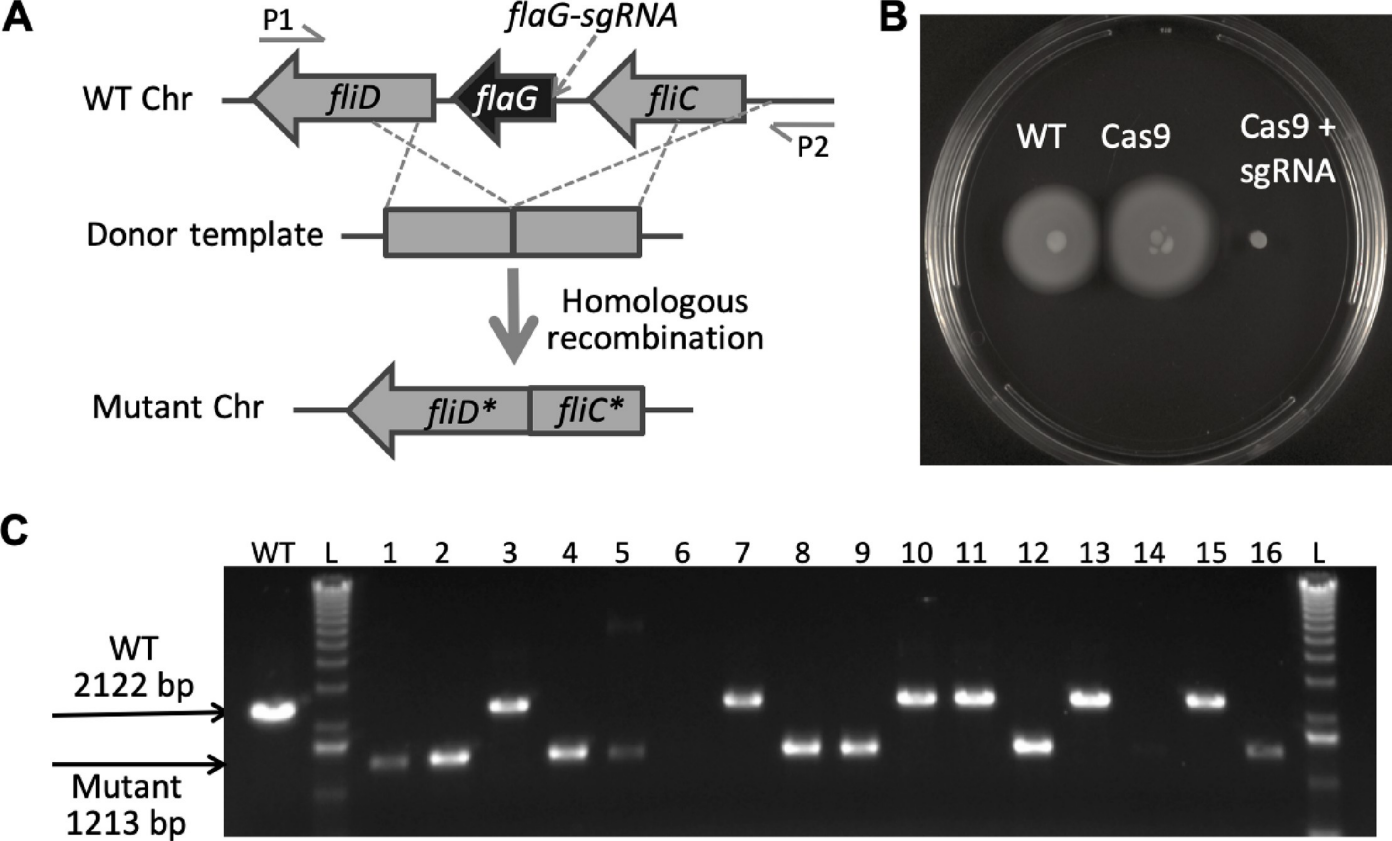
Construction of Cas9-mediated *S*. *oneidensis flaG* gene deletion mutants. (A): The editing schematic diagram and screening primers are shown for deletion of the *flaG* gene. (B): The phenotype of different *S*. *oneidensis* strains. WT, wild type. Cas9, the wild type strain harboring *Cre*, *Cas9*, *RecET* and *AprR* in the chromosome. Cas9+sgRNA, the wild type strain harboring donor template, *sgRNA*, *KanR*, *Cas9*, *RecET* and *AprR* in the chromosome. (C): PCR screening of the initial transconjugant colonies. L: DNA ladder. WT: wild type as positive control. 1–16: 16 randomly picked colonies.

The pCC1-3L and pR6K-loxWT5171-SpCas9 vectors described above are universally applied to the four bacteria. In contrast, the second accessory vector carries the sgRNA and DNA repair template and is therefore unique to each edit. We designed the second accessory vector for each target locus specifically. The second accessory vector contains donor template, sgRNA and kanamycin selection marker that are flanked by a pair of *lox* sites compatible with those flanking Cre in the landing pad. We kept the length of the donor template homologous arms in the range of 400 ~ 700 bp, and a spacer was identified specific to the targeted deletion region (see Methods for details). We took the conserved sequences 5’ to the recipient host’s 16S rRNA as promoters for the expression of the sgRNA and used 90 bp 3’ to the 5S rRNA as terminators. We introduced the second accessory vector by conjugation, and transformants were selected on plates supplemented with kanamycin, apramycin, and arabinose ensuring maintenance of the editing machinery and expression of *recET*. The intended edits were verified by colony PCR. We also confirmed the second cassette exchange by PCR amplification of the pR6K vector backbone and sgRNA payload (data not shown). Fig 2 demonstrates the edited deletion of the *flaG* gene in *S*. *oneidensis* MR-1. The screening of targeted genome edits in other three bacteria discussed above was carried out in a similar way (S3–S5 Figs).

To address any potential off-target DNA sequence changes, we used a whole genome gRNA selection software gRNA-SeqRET (Simirenko et al., manuscript in preparation) to detect any genomic sequences that have less than 5-bases mismatch with any of the selected protospacers (spacers and associated PAM). We found only one protospacer (NRPS_7, Table 1) that has one 3-bases and three 4-bases mismatches with other loci in the *P*. *luminescens* genome. We sequenced these loci and verified that none of these sites has been edited. For the rest of the 12 protospacers, there was no potential off-target sites with less than 5 bases mismatch. The details of the off-target analysis were provided in S1 File.

### Genome editing occurs at various stages of cell development in *Photorhabdus luminescens*

**Fig 3:**
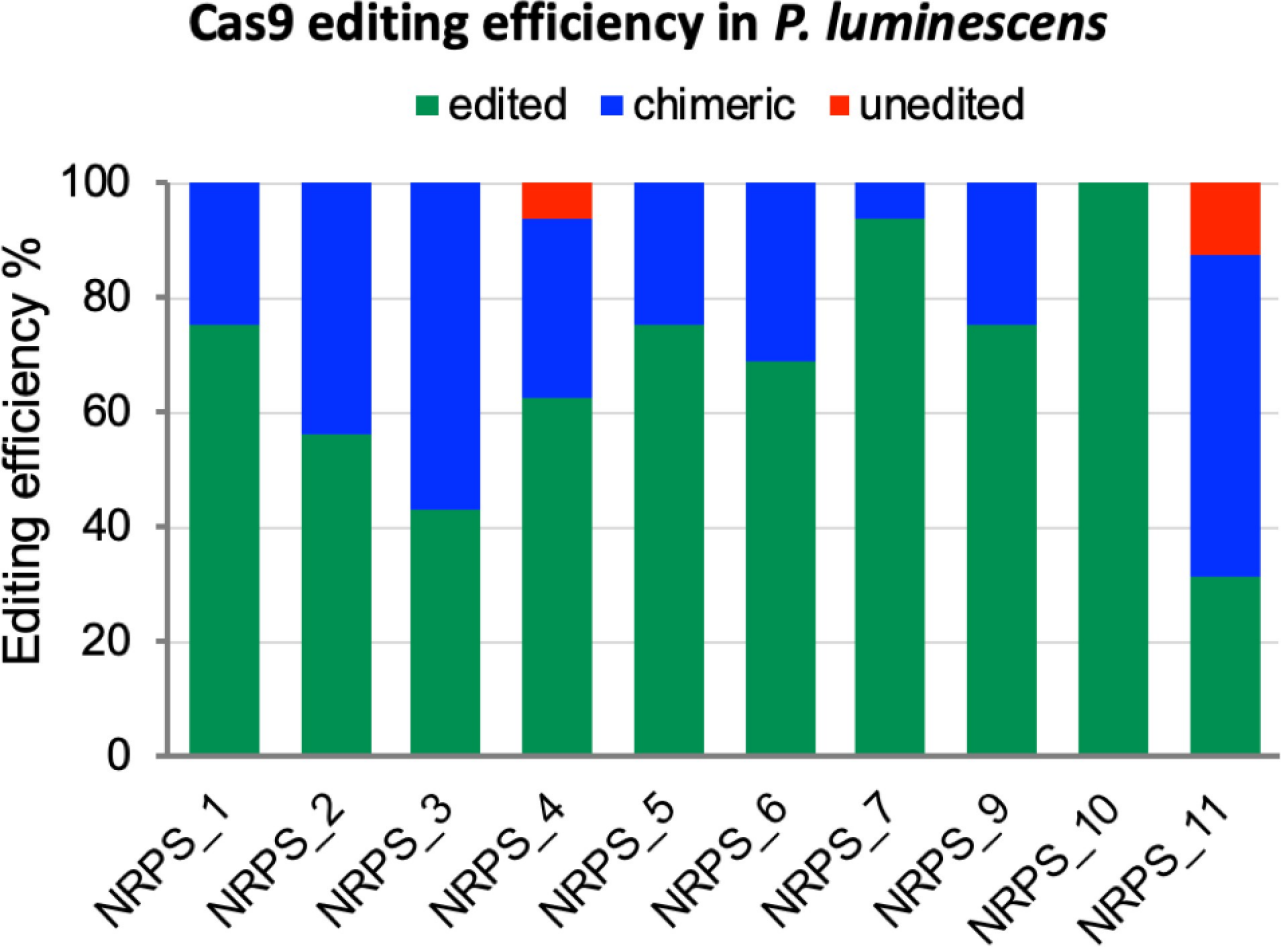
The Cas9-assisted deletion editing efficiency in *P*. *luminescens subsp*. *laumondii* TT01. Edited: colony PCR yielding an amplicon of edited cells. Chimeric: colony PCR yielding mixed amplicons with both edited and unedited cells. Unedited: PCR only yielding an amplicon of unedited cells.

The described method provides no selective pressure for the targeted editing, so a high Cas9 editing efficiency is crucial for this approach to work. To assess this, we carried out a cross-loci editing comparison in *P*. *luminescens*, and determined editing efficiencies for each target locus by colony PCR. After transforming with the second accessory vector, we selected 16 random colonies (if there were less than 16 transformants we selected all available; S5 Fig). In many cases, colonies initially contained both edited and unedited cells. However, when we re-streaked these chimeric colonies, successfully edited cells were obtained in every case (S4C Fig). This result suggests that the colony only yielding an amplicon of edited cells may still contain a very small percentage of the unedited cells, which cannot be detected by regular colony PCR screening. Therefore, we reported the editing efficiency by including the edited, chimeric, and unedited colonies based on the PCR results of transconjugants growing for 20 hours on agar plates (Fig 3). These results also suggest that more robust editing may result from routinely passaging transformant colonies under selection prior to verification. The editing efficiency ranges from 31%– 100% if only counting the edited colonies, the efficiency increases to 88%– 100% if including both edited and chimeric colonies. Overall, our data suggest that our Cas9 system could achieve high efficiency for accurate deletion editing in *P*. *luminescens*.

We have also observed different percents of chimeric colonies resulted from editing of different targets [38]. Some loci (NRPS_3 and NRPS_11) show a high percentage of chimerism, where others (NRPS_7 and NRPS_10) show a very small number or no chimeric colonies. We postulate that this difference may be due to the difference in Cas9 accessibility of different loci. Therefore, editing of different targets can occur at various stages during cell development. This chimerism phenotype can also vary from species to species, for example, we have also seen this in *A*. *salmonicida* and *P*. *simiae*, but not in *S*. *oneidensis* (Fig 2 and S3 and S4 Figs).

### Curing of landing pad and *Cas9* after genome editing in *P*. *luminescens*

**Fig 4:**
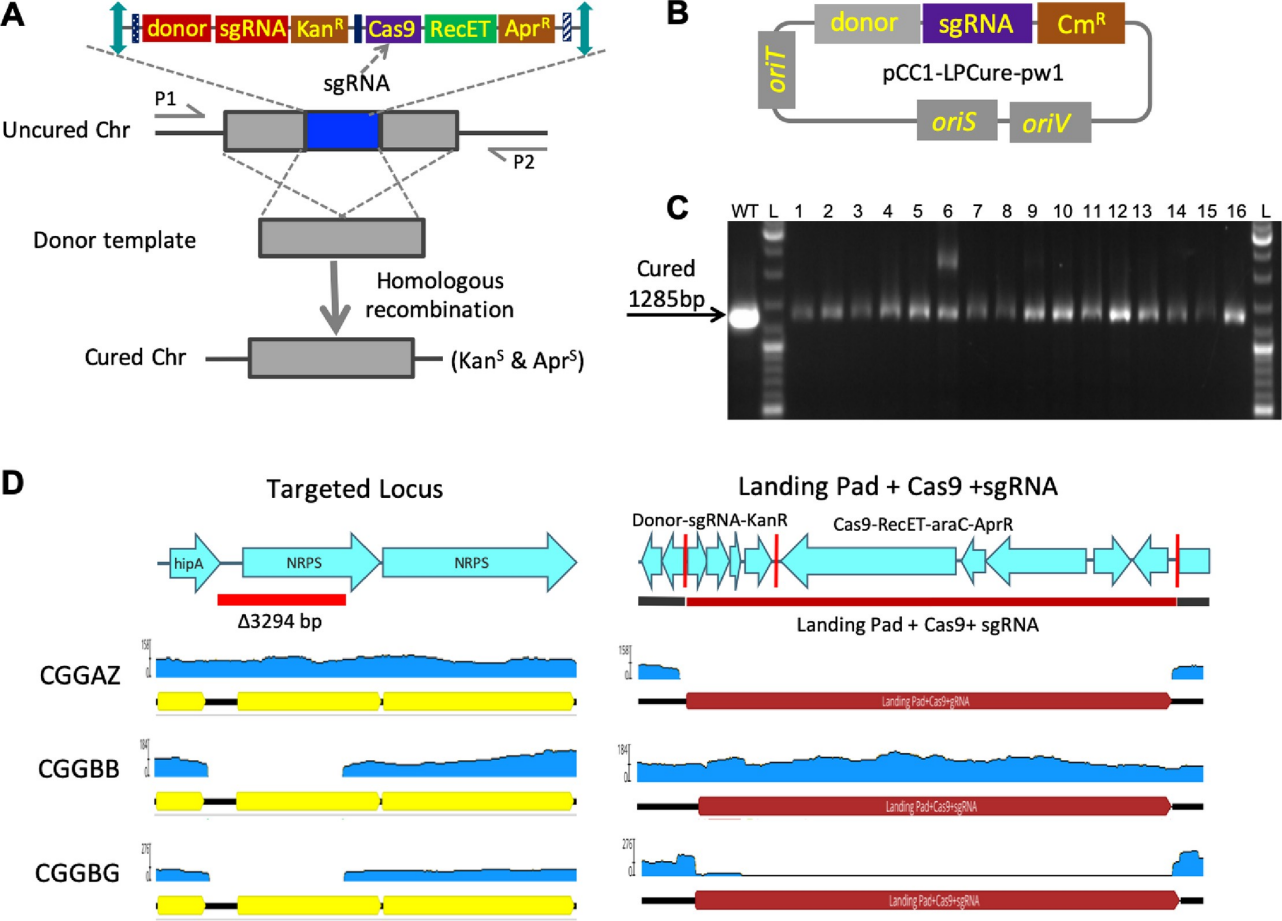
Removing the editing machinery in *P*. *luminescens*. (A): The curing schematic diagram and screening primers are shown for curing the editing machinery (not drawn to scale). (B): The structure of the curing plasmid. (C): PCR screening after curing (the larger band in isolate 6 is a non-specific amplification). L: DNA ladder. WT: wild type as positive control. 1–16: 16 randomly picked colonies. (D) The sequence alignment analysis of three strains at two specific loci on the chromosome. CGGAZ, CGGBB, and CGGBG denote as reads generated from a wild-type strain, an edited strain carrying the landing pad, *Cas9*, and sgRNA, and an edited strain with landing pad removed, respectively. The red vertical lines indicate the 3 *lox* sites in the landing pad.

To minimize the phenotypic effects of the integrated machinery, which may confound downstream experiments, an effective curing strategy to remove the editing cassettes after editing is necessary. We used the Cas9 self-targeting strategy to remove these scars in *P*. *luminescens*. To do this, we cloned a sgRNA targeting *Cas9* and the genomic DNA flanking the 3-*lox* landing pad insertion site as the repair template (S2 File) into pCC1FOS (the same backbone as pCC1-3L), to generate pCC1-LPCure-pw1 for curing. Vector with this replication origin is retained in most gammaproteobacteria under selective pressure, yet is rapidly lost in the absence of selection [39]. We again used colony PCR to confirm the curing of the landing pad with integrated editing machinery (Fig 4). We didn’t include an uncured control because the expected amplicon was too large to amplify for a regular PCR. To further verify the curing, we performed whole genome sequencing on selected cured isolates along with a wild-type *P*. *luminescens*, and a landing pad with *Cas9* inserted strain. The sequence alignment of the targeted edited site and the 3-*lox* landing pad insertion site further confirmed the successful removal of the editing machinery (Fig 4D). The cells also lost the curing plasmid after successive culturing without the presence of antibiotics.

## Discussion

We developed a chromosomally integrated CRISPR-Cas9 system that we have demonstrated to be easily applicable to several gammaproteobacterial species. The method involves the insertion of a Cre-*lox*-containing landing pad into the chromosome by transposon integration. We incorporated three mutually exclusive *lox* sites facilitating two sequential rounds of Cre-assisted cassette exchange. The first round of RMCE brings in *Cas9* and *RecET* to the chromosome. The second round of RMCE brings in the sgRNA and donor template. By integrating all the components into the chromosome, we have demonstrated (1) genome editing in bacterial cells without a need for a replicative plasmid system, and (2) that the integrated landing pad and *Cas9* can be removed to achieve scarless editing of bacteria genome. To the best of our knowledge, this work constitutes the first report of Cas9-based genome editing system for *A*. *salmonicida*, *P*. *simiae* and *P*. *luminescens*.

The system takes advantage of well-characterized genetic parts that are known to be functional in taxonomically diverse bacterial species, likely expanding the utility of our approach with minor modifications to other classes beyond the present study. Those key elements include the mariner transposon system, Cre-*lox* recombinase system, CRISPR-Cas9, RecET orthologous pair for DNA double strand break repair, and several antibiotic selection markers. We demonstrated the successful application of this approach to species from four taxonomically diverse orders (Alteromonadales, Pseudomonadales, Enterobacterales and Aeromonadales) within gammaproteobacteria, which underscores the likely broader applicability of our approach to many or perhaps most of this lineage.

It is certainly possible were we to place the *Cre*, the *Cas9* and *recET* recombinase genes outside the *lox* sites, and only use the *lox* sites for exchanging the sgRNA and donor template as a simplified, scaled down version of our design, could work. However, the 3-*lox* landing pad offers more versatility than either 2-*lox* or 1-*lox* landing pads; for example, once the landing pad has integrated, different editing machinery recognizing distinct PAM sequences can easily be exchanged as desired within the same genome. The versatility offered by the 3-*lox* landing pad is especially adaptable in cases where more than one type of editing enzymes such as dCas9 and/or aCas9 are desired in addition to the regular Cas9. Consequently, and with only minor changes to our outlined workflow, complex engineering can be achieved.

Multiple editing can be achieved by performing sequential editing. There are two options enabling sequential editing using the 3-*lox* system. First, we can move the Cre recombinase gene to outside the *lox* sites (between IR and *lox*2272 for example; Fig 1). This way we can continue using the 2^nd^ *lox* exchange site (between *lox*2272 and *lox*P) for swapping in new editing targets carrying a different selectable marker. Secondly, including only a single *lox* site on the 2^nd^ accessory vector (which carries sgRNA and donor DNA template), as the RMCE will insert the entire 2^nd^ accessory vector into the landing pad. Under appropriate antibiotic selection, the sgRNA and donor template should remain inside the landing pad despite being flanked by two identical *lox* sites. After editing, the entire 2^nd^ accessory vector can be removed again by Cre-*lox* recombination when selective pressure is lifted. Subsequent to this event, cells carrying only the Cre and Cas9 will be primed for additional editing.

We observed chimeric phenotypes in the editing of three out of four organisms, indicating that chimerism is rather common (at least in the organisms used in this study). A chimeric colony is formed when the editing initiates after the first few cell divisions. This result suggests that the cells can tolerate the presence of Cas9 and sgRNA throughout the cell divisions. An open question is why editing does not always occur at the same stage and how some cells can delay or skip the editing until a later stage. We plan to take advantage of this delay editing characteristics and insert the landing pad curing sgRNA and donor template into the 2^nd^ *lox* exchange site. The delay editing would then allow us to select colonies for the insertion of the curing sgRNA and donor template first, then remove the antibiotics selection enabling the curing of the whole landing pad.

In this study the genetic parts (promoters, coding genes, and selectable markers) used in the 3-*lox* landing pad are functional in bacteria across the gammaproteobacterial classes. We believe this strategy has significant potential for genome engineering in bacteria of other phyla if we can tailor those genetic parts that are necessary to work in another phylum. Given the broad functionality of phage-derived genetic elements [40], one future endeavor could be to incorporate a T7 RNA polymerase driven by a host-specific promoter. The host-specific promoter will ensure the expression of the T7 RNA polymerase thus ensuring the expression of the key components.

Ultimately, the goal of this work was to provide a generalizable strategy for domesticating bacteria through genome editing, thereby accelerating our ability to engineer microbes in the microbiome era. We demonstrated here the feasibility to apply a single system to achieve precise genome editing in multiple Proteobacterial species. We predict that the list of bacterial species amenable to this genome editing method will continue to expand considering the solid cross-species functionality of all the key components of this method. We anticipate that our work will inspire future genetic tool development to overcome the cross-species challenge.

## Materials and methods

### Bacterial strains and growth conditions

The bacterial strains used and generated in this study are listed in S1 and S2 Tables. *Escherichia coli* TOP10 cells were used for plasmid assembly. *E*. *coli* BW29427 (aka WM3064) was used as a conjugal donor strain. All growth media were purchased from Teknova (www.teknova.com), and other chemicals were purchased from Sigma-Aldrich. *E*. *coli* was grown in LB medium at 30°C supplemented with antibiotics as needed: 50 μg/ml carbenicillin, 20 μg/ml chloramphenicol, 50 μg/ml kanamycin, 50 μg/ml apramycin. To culture *E*. *coli* BW29427, a final concentration of 300 μM diaminopimelic acid (DAP) was supplemented in LB. The culturing conditions of the edited strains are listed in S3 Table.

### DNA manipulation

All primers were ordered from Invitrogen (Thermo Fisher Scientific). Synthetic DNA fragments were ordered either from Integrated DNA Technologies (IDT) or from Thermo Fisher Scientific. PCR and Gibson assembly were performed using Q5 hot start DNA polymerase and Gibson Assembly HiFi HC 1-step kit (SHI-DNA, Inc. CA), respectively according to the manufacturer’s directions. Plasmid DNA was isolated using the QIAprep spin miniprep kit and genomic DNA was extracted using Wizard genomic DNA purification kit (Promega). We used the Zymo clean gel DNA recovery kit to further purify DNA (Zymo Research).

### Plasmid construction

The sequence maps of the vectors are listed in S2 File. The primers and PCR template, including gBLOCKs, were listed in S6 Table. Constructs were sequence-verified by either Sanger Sequencing or Pacific Biosciences Sequencing.

### The 3-*lox* landing pad vector pCC1-3L

The landing pad vector was constructed with five DNA fragments comprising (1) *parC*, *parB*, *parA*, *repE*, *oriS*, *oriV*, *redF*, and a Mariner *IR*, (2) *lox*2272, (3) *Cre*, (4) *lox*P, and (5) *KanR*, *lox*5171, Mariner *IR*, *oriT* and Mariner transposase. Fragments 1, 3 and 5 were PCR amplified from pW17 (Wang et al., 2019), and fragments 2 and 4 were PCR amplified from a synthetic DNA fragment, 2lox.gBLOCK (S6 Table). Fragments 2 to 5 were then fused together by overlapping PCR, and then assembled with fragment 1 by Gibson Assembly. The sequences of the 3 *lox* sites are listed in S4 Table.

### The 1^st^ accessory vector pR6K-loxWT5171-SpCas9

The *Cas9* was first PCR amplified from *Streptococcus pyogenes* genomic DNA and cloned into the *Nco*I site of the pKD46 vector (The Coli Genetic Stock Center) by Gibson Assembly. The resulted pKD46-SpCas9 plasmid was then used as the template to amplify two fragments, one containing the tL3-Cas9 and the other containing the araC-pBAD cassette. These two fragments were then Gibson assembled together with four other PCR amplified fragments to form the pR6K-loxWT5171-SpCas9R4 plasmid. Of the four fragments, two contain the *oriT* and *lox*P amplified from pW17, one contains the *AprR-lox5171-R6K* cassette amplified from the pW34 plasmid (Wang et al. 2019), and one synthetic DNA fragment contains the *recET* recombinase gene (RecET.gBLOCK).

### The 2^nd^ accessory vectors

For cloning sgRNA and donor template, we first constructed a vector pR6K-lox2272WT by Gibson Assembly using 4 PCR amplified DNA fragments comprising (1) *R6K* and *oriT*, (2) *lox*2272, (3) *KanR*, and (4) *lox*P. These fragments were amplified from either pCC1-3L or pR6K-loxWT5171-SpCas9 plasmids (S6 Table).

The pR6K-lox2272WT vector was then used to clone the sgRNA scaffolds in two different approaches. In cases of *S*. *oneidensis*, *P*. *simiae*, and *P*. *luminescens*, we clone a synthetic DNA fragment containing either the 16S promoter-spacer-sgRNA scaffold-5S terminator cassette or just the spacer-sgRNA scaffold-5S terminator cassette with a PCR amplified 16S promoter into the *Zra*I site of the pR6K-lox2272WT. The single spacer of these plasmids was then replaced with a linker sequence containing two *Bsa*I sites by PCR to allow cloning of additional spacers. In case of *A*. *salmonicida*, we PCR amplified the 16S promoter and 5S terminator from genomic DNA, and an sgRNA scaffold from synthetic DNA, and Gibson assembled them into the *Zra*I site of the pR6K-lox2272WT. In the design of PCR primers, we also incorporated two *Bsa*I sites 5’ to the sgRNA scaffold to allow cloning of spacers (S6 Fig). The primers and synthetic DNA used in the construction of these sgRNA scaffold containing vectors are listed in S6 Table.

The sgRNA targets were synthesized as forward and reverse oligos (S6 Table). These oligos were annealed to form 4-base overhangs on either side of the spacers, which can then be ligated into the *Bsa*I sites of these organism specific sgRNA scaffold containing vectors. The spacer containing vectors were then used to clone the donor template at the unique *Sac*I site of the vectors. The donor templates containing upstream and downstream regions of the targeted deletion sites were amplified by PCR using the primers listed in S6 Table.

### The curing vector

The curing plasmid for *P*. *luminescens* was constructed in two steps. First, we constructed the intermediate vector pCC1-LPCure, by Gibson Assembly of 5 PCR amplified DNA fragments comprising (1) parC, parB, parA, partial repE, (2) partial repE, oriS (ori2), oriV, redF, and Cat, (3) oriT, (4) 16S promoter, and (5) sgRNA scaffold and 5S terminator. Then two primers (Cas9-sgRNA.fwd and Cas9-sgRNA.rev) were annealed together to form the spacer targeting the Cas9 and cloned into *AarI* digested pCC1-LPCure. The resulted plasmid was then digested with *SacI* to clone in the PCR amplified donor template (S6 Table) by Gibson Assembly. The final curing plasmid is pCC1-LPCure-pw1.

### Conjugation transformation procedures

Plasmids were introduced to the target organisms by conjugation. Overnight cultures of both donor and recipient strain cells were diluted into fresh medium (1:100 ratio) and grew until they had reached a density of 1 at 600nm. The donor cells were washed with fresh medium without antibiotics three times, and the recipient cells with different ratios (S3 Table) were mixed together, pelleted and resuspended to the final volume of 60 μL with LB liquid medium supplemented with DAP. The resuspension was spotted onto a MF-Millipore 0.45 μm gravimetric analysis membrane filter, and incubated overnight on an LB agar plate supplemented with DAP at 30°C. The next day, the conjugation mixture was scraped off from the membrane and resuspended into 1 mL fresh LB, different serial dilutions were plated onto LB agar plate supplemented with appropriate antibiotics but without DAP for the counter selection of the *E*. *coli* donor cells. Plates were incubated for 1~3 days.

### Mapping the chromosomal integration location of the landing pad

We mapped the landing pad insertion site using inverse PCR (Martin and Mohn, 2002). We used a 4-base cutter enzyme (either *HpyCH4IV* or *NlaIII*) to digest extracted chromosomal DNA of kanamycin resistant transformants, followed by a ligation reaction to circularize the fragments. Specific regions of interest were PCR amplified using primers (S5 Table) designed to flank the integrated transposon and sequence verified.

### Selection of spacer for sgRNA

We manually selected the spacer for the sgRNA for each targeted gene using the following criteria. (i) The spacer sequence is located in the upstream regulatory or coding sequence region; (ii) it must immediately precede a 5’-NGG protospacer adjacent motif (PAM); (iii) this spacer does not match with any other loci in the genome; (iv) the GC content of the spacer is between 40~60%; (v) there is no inverted repeats or direct repeats. We also used an in house gRNA selection tool, gRNA-SeqRET (Simirenko et al., manuscript in preparation) to identify potential off-target loci. For any possible off-target sequences with less than 5 bases mismatch, we designed primers and carried out sequencing analysis of the amplicons.

### Validation of CRISPR-Cas9 edited mutants

We picked 16 colonies that were both apramycin and kanamycin resistant, and PCR amplified and sequencing validated each targeted region. The primers used for screening were listed in S6 Table.

### Sequencing verification of the removal of the editing machinery in *P*. *luminescens*

Genomic DNA from a wild-type *P*. *luminescens subsp*. *laumondii* TT01, an edited NRPS_1 deleted strain carrying the landing pad and CRISPR-Cas9 machinery, and an edited NRPS_1 deleted strain with the landing pad removed were purified and sequenced using the PacBio RSII sequencer according to the manufacture protocol (Pacific Biosciences Inc. CA). *P*. *luminescens subsp*. *laumondii* TTO1 (GenBank: BX470251.1) that contained the insertion of Landing Pad was used as a reference for mapping. The insertion site of the landing pad was based on previous verification. Mapping was performed using cleaned-up PacBio subreads with Minimap2 [41]. SAMtools was used to summarize mapping results and generate bam files for genome browser [42].

## Supporting information

S1 Fig The two Cre-*lox* Recombinase-Mediated Cassette Exchange (RMCE) events (not drawn to scale). (TIF)

S2 Fig Growth curve of the wild type strain and its landing pad insertion derivative for four species. (A-D): *A*. *salmonicida subsp*. *Pectinolytica* 34mel, *P*. *luminescens* subsp. *laumondi*i TT01, *P*. *simiae* WCS417r and *Shewanella oneidensis* MR-1, respectively. The growth curve was carried out in LB medium at 30°C with biological duplicates.
(TIF)

S3 Fig Construction of Cas9-mediated *A*. *salmonicida hpd* gene deletion mutants. (A): The editing schematic diagram and screening primers are shown for *hpd* gene deletion (Asalp_21790) (not drawn to scale). (B): PCR screening of the initial transconjugant colonies. L: DNA ladder. WT: wild type as positive control. 1–16: 16 randomly picked colonies. (C) The phenotype of different *A*. *salmonicida* strains. WT, wild type. Cas9+sgRNA, the wild type strain harboring donor template, *sgRNA*, *KanR*, *Cas9*, *RecET* and *AprR* in the chromosome. Cas9, the wild type strain harboring *Cre*, *Cas9*, *RecET* and *AprR* in the chromosome.
(TIF)

S4 Fig Construction of Cas9-mediated *P*. *simiae* thioesterase encoding gene deletion mutants. (A): The editing schematic diagram and screening primers are shown for deletion of the thioesterase encoding gene (PS417_19550) (not drawn to scale). (B): PCR screening of the initial transconjugant colonies. (C): PCR screening of the colonies streaked out from an initial chimeric transconjugant colonies. L: DNA ladder. WT: wild type as positive control. 1–16: 16 randomly picked colonies.
(TIF)

S5 Fig Construction of Cas9-mediated *P*. *luminescens* deletion mutants for genes encoding predicted non-ribosomal peptide/polyketide synthases (NRPS). L: DNA ladder. WT: wild type as positive control. 1–16: 16 randomly picked colonies. For the gene deletion of NRPS_3, we only got total 7 kanamycin and apramycin resistant colonies from 3 different conjugation experiments.
(TIF)

S6 Fig Schematic diagram for the secondary accessory vector construction (not drawn to scale). (TIF)

S1 Table Bacterial strains used in this study. (XLSX)

S2 Table Strain selected with landing pad transposon insertion. (XLSX)

S3 Table Culturing conditions for bacterial strains. (XLSX)

S4 Table The sequence of the *lox* sites used in this study. (XLSX)

S5 Table Primers used for inverse PCR. (XLSX)

S6 Table Primers and gBLOCKs used for vector construction and mutant screening. (XLSX)

S1 File (ZIP)

S2 File (ZIP)

S1 Raw images (PDF)
