# Supplementary figures and images for: Bacterial genome editing by coupling Cre-lox and CRISPR-Cas9 systems
Source: PLoS One. 2020 Nov 4;15(11):e0241867. doi: 10.1371/journal.pone.0241867 (PMC7641437; doi:10.1371/journal.pone.0241867)

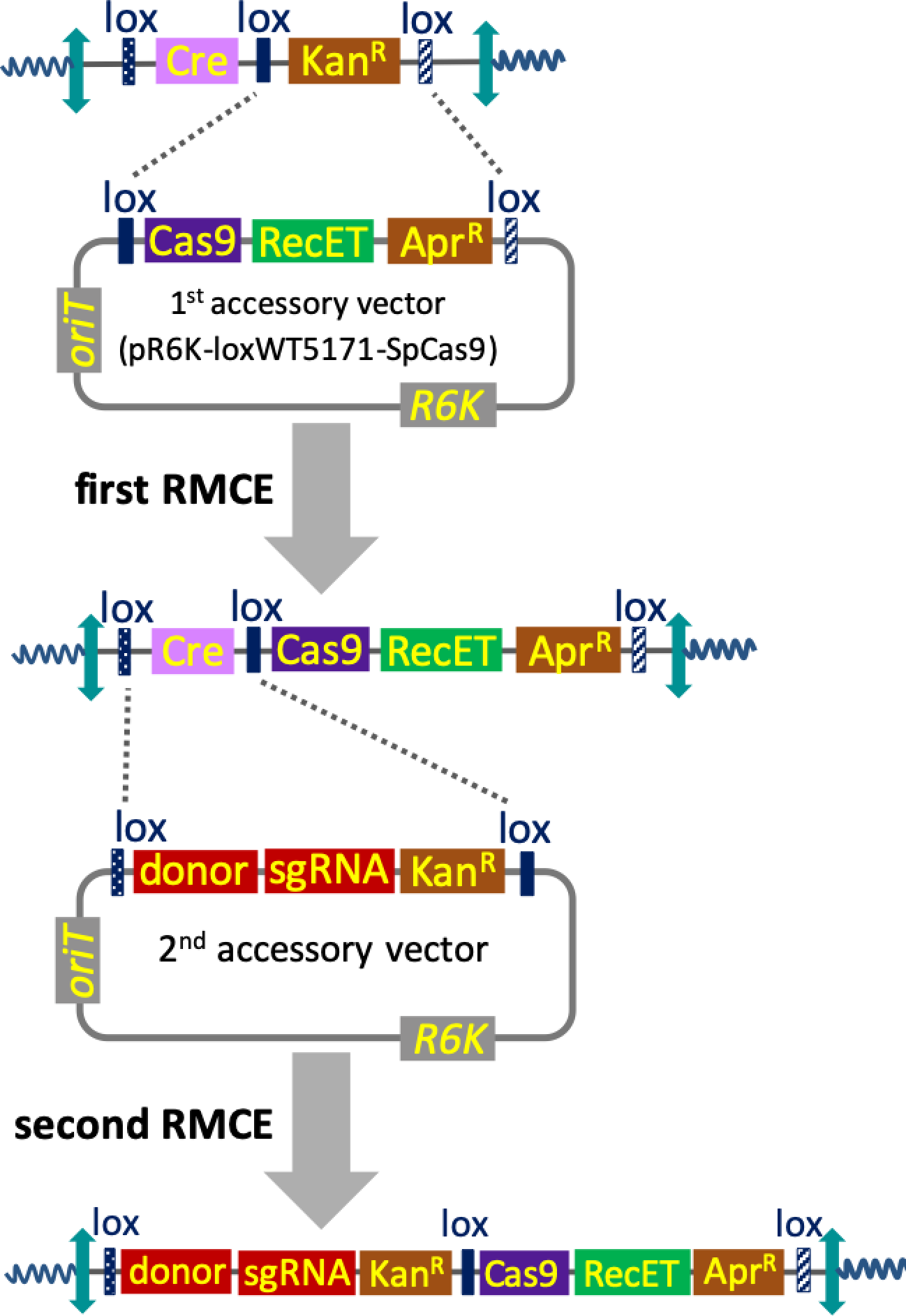

Supplement: S1 Fig — (TIF) [file pone.0241867.s001.tif]

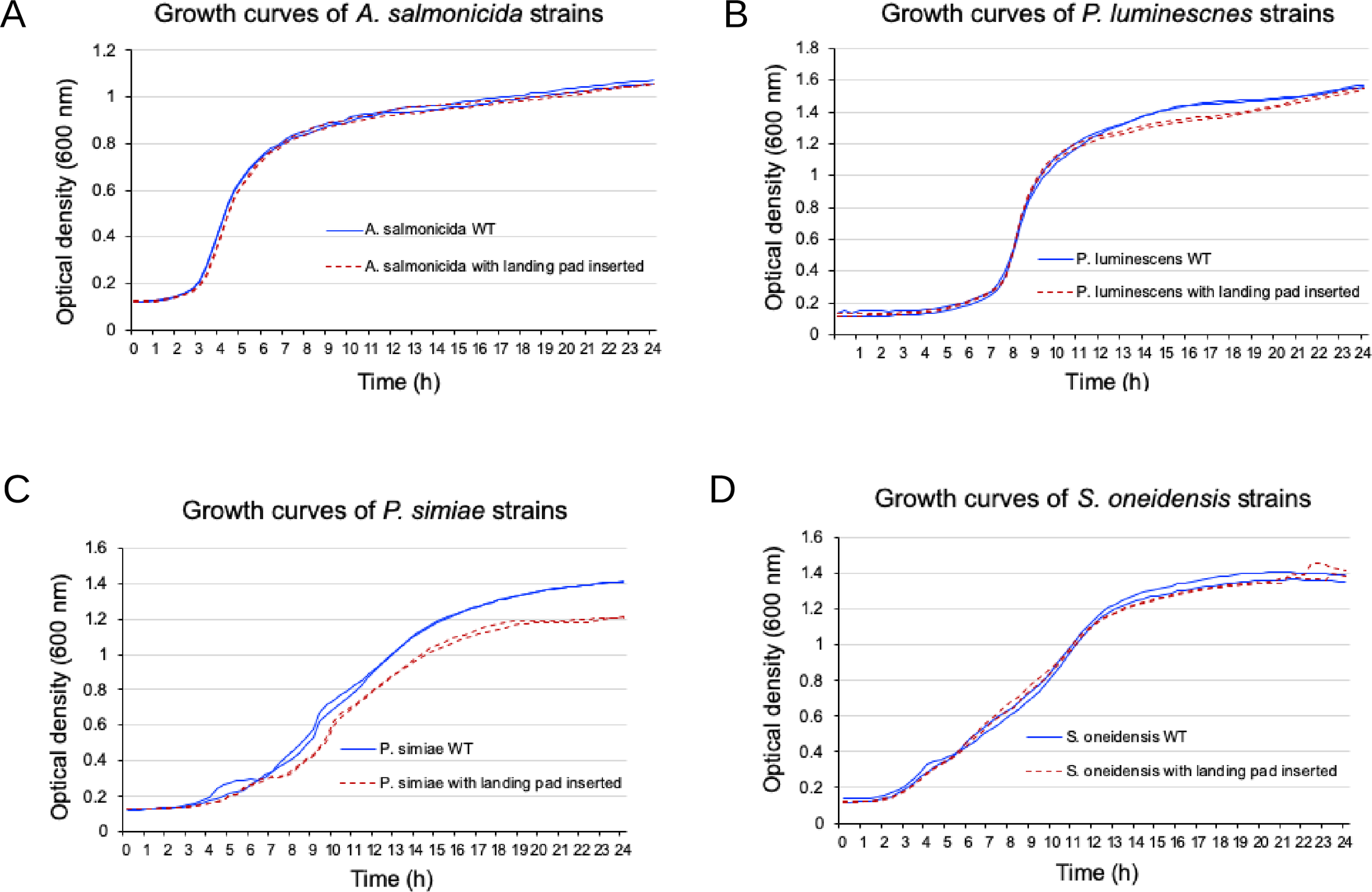

Supplement: S2 Fig — (A-D): A. salmonicida subsp. Pectinolytica 34mel, P. luminescens subsp. laumondii TT01, P. simiae WCS417r and Shewanella oneidensis MR-1, respectively. The growth curve was carried out in LB medium at 30°C with biological duplicates. (TIF) [file pone.0241867.s002.tif]

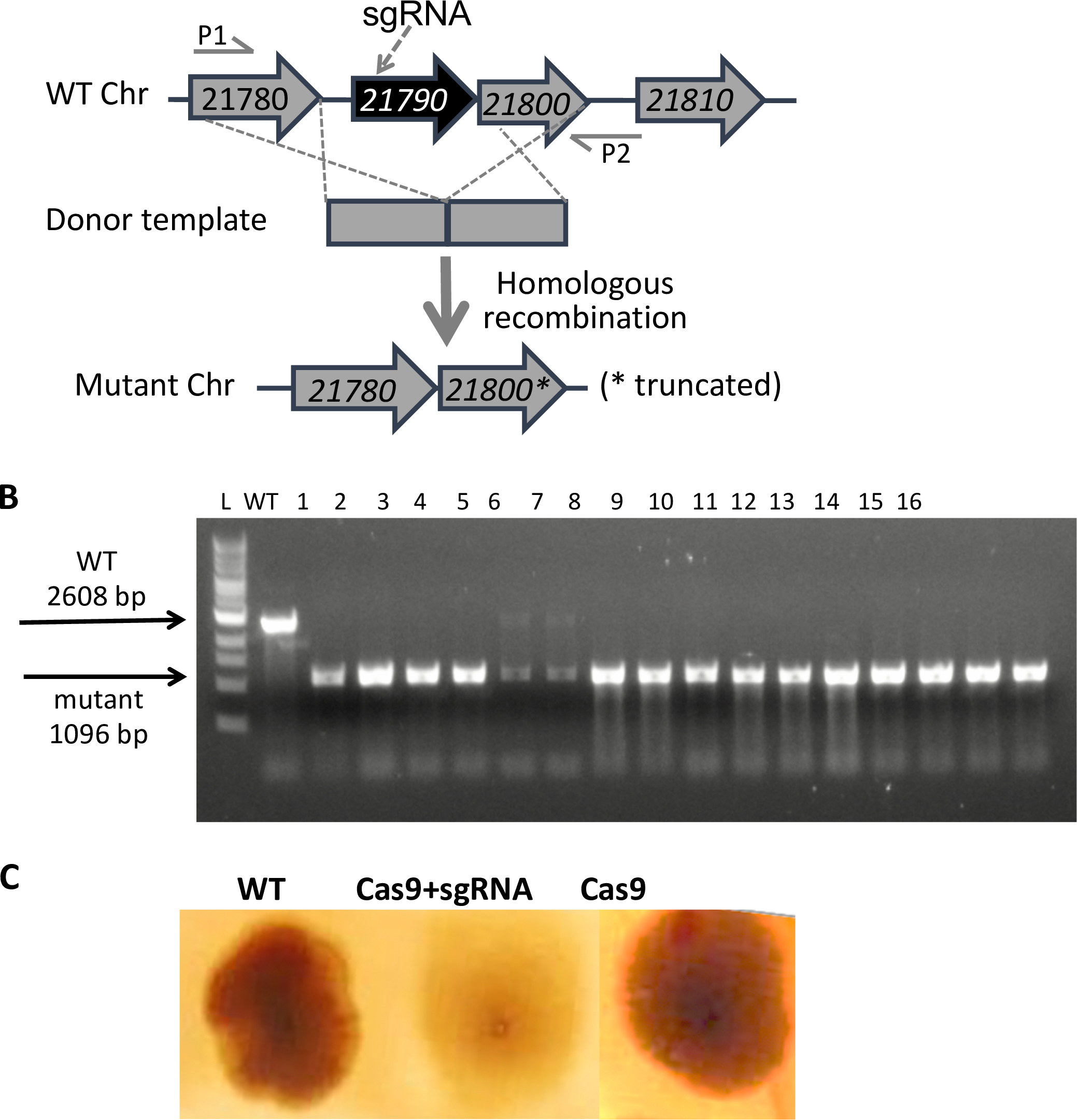

Supplement: S3 Fig — (A): The editing schematic diagram and screening primers are shown for hpd gene deletion (Asalp_21790) (not drawn to scale). (B): PCR screening of the initial transconjugant colonies. L: DNA ladder. WT: wild type as positive control. 1–16: 16 randomly picked colonies. (C) The phenotype of different A. salmonicida strains. WT, wild type. Cas9+sgRNA, the wild type strain harboring donor template, sgRNA, KanR, Cas9, RecET and AprR in the chromosome. Cas9, the wild type strain harboring Cre, Cas9, RecET and AprR in the chromosome. (TIF) [file pone.0241867.s003.tif]

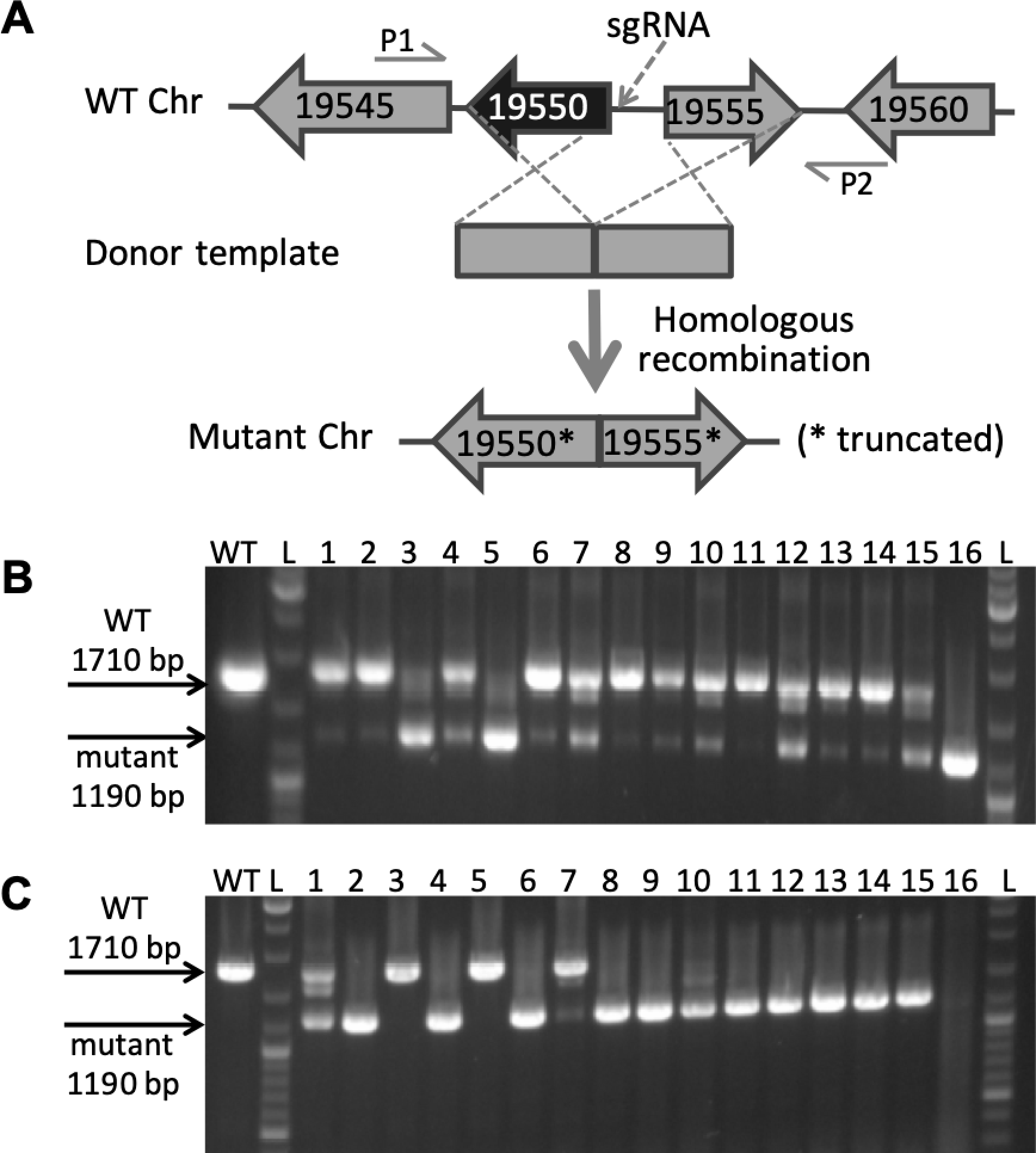

Supplement: S4 Fig — (A): The editing schematic diagram and screening primers are shown for deletion of the thioesterase encoding gene (PS417_19550) (not drawn to scale). (B): PCR screening of the initial transconjugant colonies. (C): PCR screening of the colonies streaked out from an initial chimeric transconjugant colonies. L: DNA ladder. WT: wild type as positive control. 1–16: 16 randomly picked colonies. (TIF) [file pone.0241867.s004.tif]

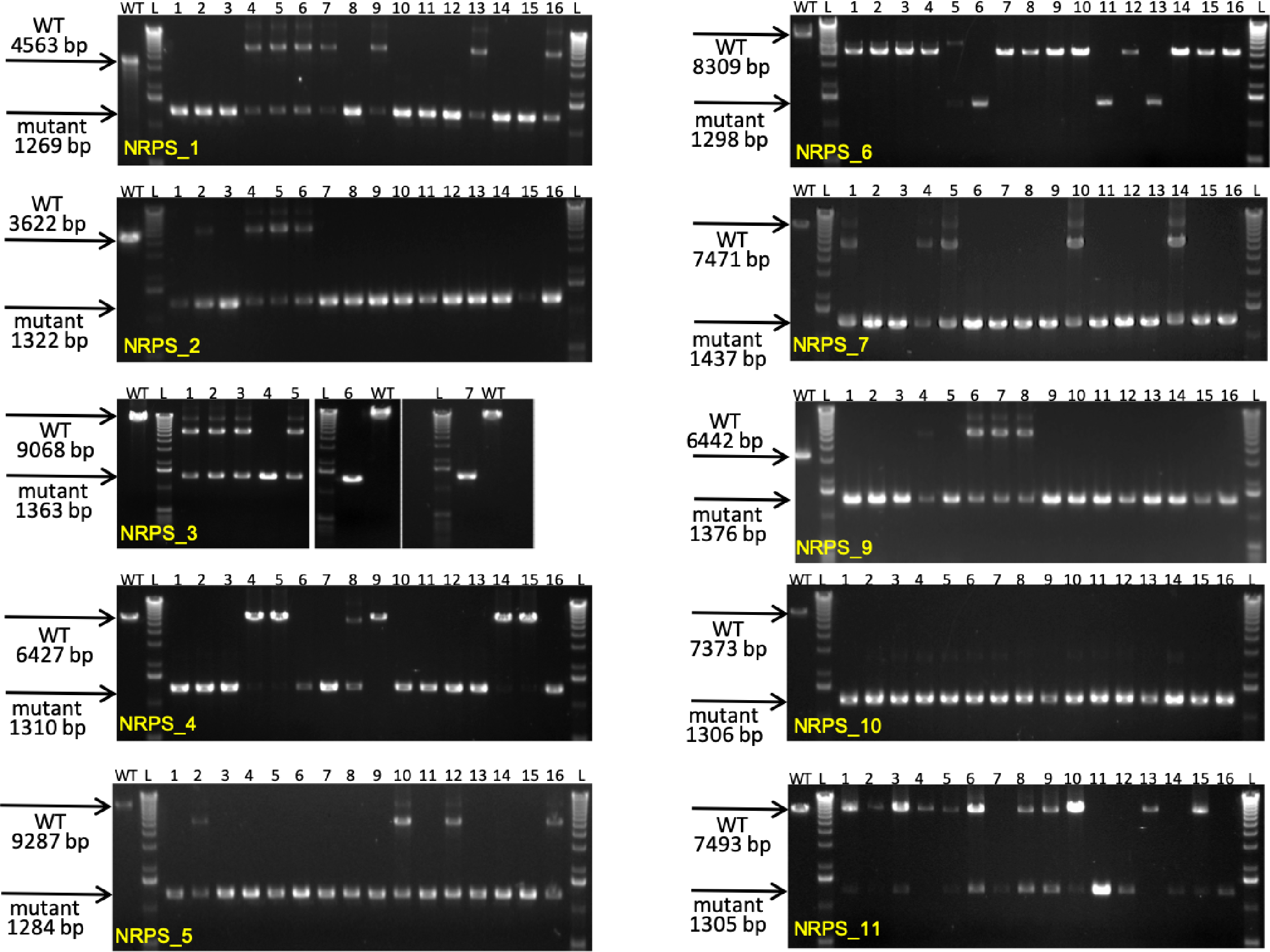

Supplement: S5 Fig — L: DNA ladder. WT: wild type as positive control. 1–16: 16 randomly picked colonies. For the gene deletion of NRPS_3, we only got total 7 kanamycin and apramycin resistant colonies from 3 different conjugation experiments. (TIF) [file pone.0241867.s005.tif]

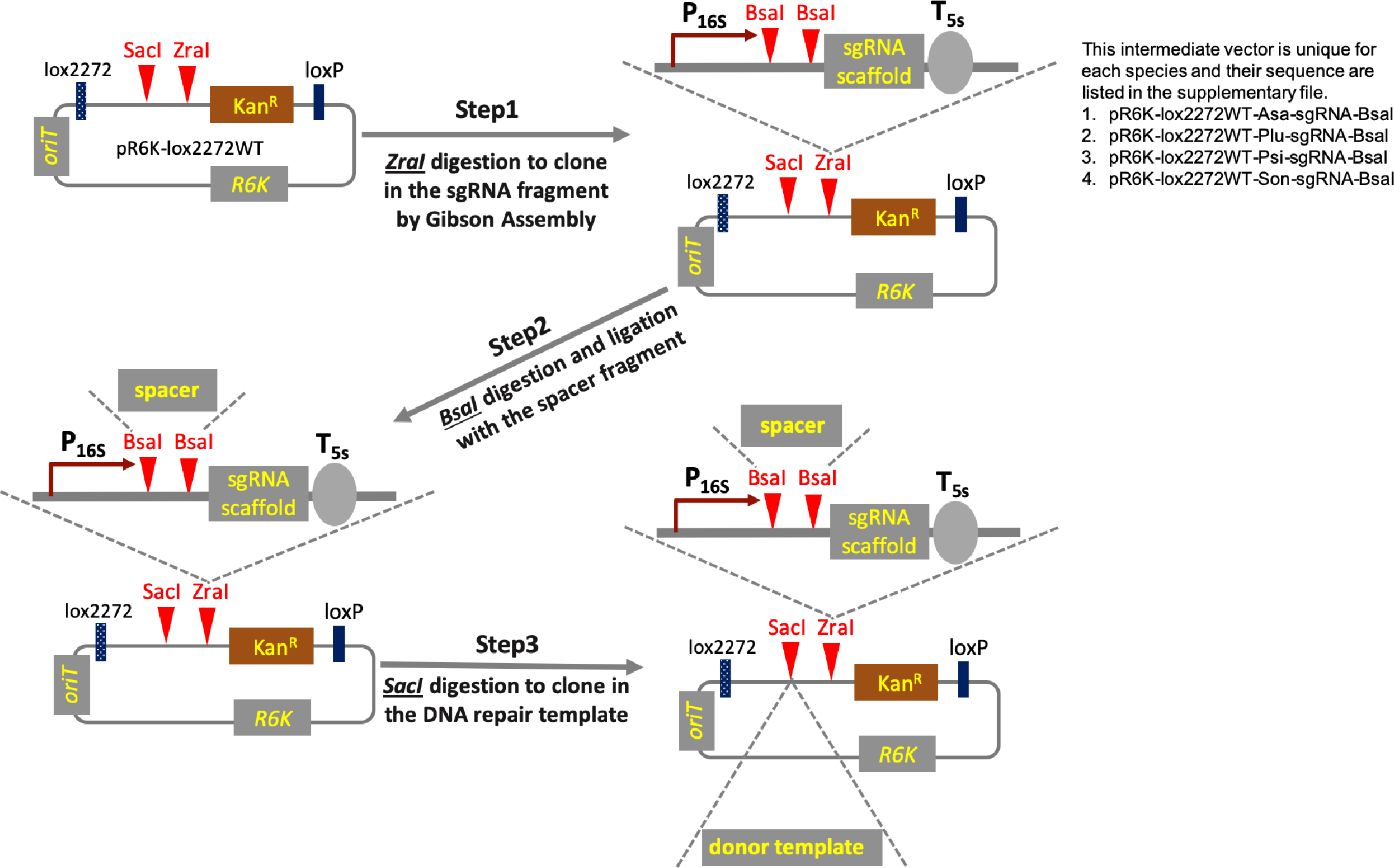

Supplement: S6 Fig — (TIF) [file pone.0241867.s006.tif]
