# Supplementary material for: Bacterial genome editing by coupling Cre-lox and CRISPR-Cas9 systems
Source: PLoS One. 2020 Nov 4;15(11):e0241867. doi: 10.1371/journal.pone.0241867 (PMC7641437; doi:10.1371/journal.pone.0241867)
Supplement: S1 File — (ZIP) [file pone.0241867.s013.zip › off-target-analysis-Archive/3-lox-PlosOne_Supp_File_1_off-target report.docx]

I searched all 13 editing targets in 4 organisms for possible off-target sequences. I had to use the target spacer sequences plus their flanking sequences (see below) to search for (1) increasing sensitivity, and (2) adding some potential spacers next to the spacers we used for comparison purposes. Below is the summary of the search. Each spacer search has an individual excel file that contains the details of hits (zipped files). The blue font sequences are repetitive sequences in the genome. Only one target (in green fonts) contains less than 5 bases mismatch, which we can design primers to detect if any off-target changes.

Organism (genbank) - spacer sequence - PAM

Pseudomonas simiae WCS417 (NZ_CP007637.1) - GCATACAGCGTGTTTCAGTT - TGG (no off-target sequences with less than 5 mismatch)

Sequence used for searching

TCGGGTCGGCGCACTCATCGAGTGACGACTCCAAACTGAAACACGCTGTATGCACGATAAAAACCCATTTCGTCCTCCTGCGG

*Aeromonas salmonicida subsp. Pectinolytica* 34mel (NZ_CP022426.1) - AGAACCCAGCGATGCGTAAC - TGG (no off-target sequences with less than 5 mismatch)

Sequence used for searching

ATTTCCAGGCACTGTTCGAATCCATTGAACTGGACCAGATCCGCCGTGGTGTACTGCATGGAGAACCCAGCGATGCGTAACTGGATAAGCTTTCCCCATCGGGAGGGAACTCACTCCCGTCAGGCGCATGCCGACCTGCCAGAG

Shewanella oneidensis MR-1 (NC_004347.2) - ACAAATGCTTATGATGGTGAGGTATAGCTA - TGG (no off-target sequences with less than 5 mismatch)

Sequence used for searching

TTGAGCCAAATCAACACGGTTTGAGACCTGCATTGATGTGCTTGCTATATTGATATCCATAGCTATACCTCACCATCATAAGCATTTGTCTTTTACAGCCTAACAAACACTATAACTTAATGACCATTTATGTGGCAAAGGGAG

*Photorhabdus luminescens* subsp. *laumondi*i TT01 (NC_005126.1) -

target 1: TGCAGATTTATATTAATAGC - AGG (no off-target sequences with less than 5 mismatch)

Sequence used for searching

TGCCATTATGTCCAGTCAGTAAAAAGAGAATATCAGTTATTTTTGTGATTAATGTTTTTTAGATGCAGATTTATATTAATAGCAGGTAAATAGTTAATTGATAAATAGAATATTCCTATTATATTTTTAAATGGTTATTAACTTAGGTG

target 2: TAAATAGTTATGATAACTAT - AGG (no off-target sequences with less than 5 mismatch)

Sequence used for searching

ATATTTTATGAATACTTGATTAATTAATGATATTATCCTCAGCTCAATGAAATAGAAAGTAATAATAAATAGTTATGATAACTATAGGATTAACACACTTAAAATAACCAATAAATTTATTGAGGTAATAAATGAAAGATAACATTGCT

target 3: GTATACATATCCAAGTTACT - TGG (no off-target sequences with less than 5 mismatch)

Sequence used for searching

ATAGATAACGATGTGACCGGGGTGAGTGAGTGCAGCCAACAAAGAGGCAACTTGAAAGATAACGGGTATACATATCCAAGTTACTTGGATATACGGTTTAAATCTATTTTAAGAGTATCGAAATAGTGCCAGATATTTCGAATTCGTGC

target 4: ATCGAGATATTCTTATTTAT - AGG (no off-target sequences with less than 5 mismatch)

Sequence used for searching

TCCCTTGCCGCCGCGATGCATCTTGAAATCCATAGGGTATATATTTAATGATTTTAAAAAACACCTATAAATAAGAATATCTCGATTTAGCATAGAACAACACTGCCATGCTTTTTGTCGATCTACAAAGTCAATCAATCAATTTCTGA

target 5: CCCTATTCCGTTAGCCCTGA - TGG (no off-target sequences with less than 5 mismatch)

Sequence used for searching

TAAATATAACGCAATTTATCATTAATGAGAATCATTATCAATAGTACGTAATGCGCTACTTTTGTCCCTATTCCGTTAGCCCTGATGGAAGACTATCGATAATCTCCCGTTATGTTTTTTGTTTTACCACACCTAATTTTCGTAAAACA

target 6: TACCCGTTTTTATTTCACCG - AGG (no off-target sequences with less than 5 mismatch)

Sequence used for searching

TTGGCAACTGTCATTCTTGACAGTCGTCACAAGTGTTTTTTCTTAACTGTTGTCCCACCCACCCTCGGTGAAATAAAAACGGGTACGTTAAGCATTAATTAAAATACTCAGGAAATGCTTTCTCTCTGGAATTGGATGTAAATAACTTA

target 7: AGTGCCGTTCCGTCATCTGG - TGG (one 3bp mismatch, three 4bp mismatch)

Sequence used for searching

CGCCAACATATCAGTGAAGAAACGGGTATGCGCTTCCTGGCTCACCCCAAGCCGAGCTTCAGCCACCAGATGACGGAACGGCACTGGCGCAGGCAGGCGATCCCCTTGCCCGGAAAAATAGGCCTGCACTTCACGGTGCATCACTTCCA

target 8: CGGTATCTAAGTCTCTTGGA - TGG (no off-target sequences with less than 5 mismatch)

Sequence used for searching

AGTCATCACTAATGTTTCTCTCCTACTTCTTGCCCAAAAATAAAAATAGACACATCTGCTATCCATCCAAGAGACTTAGATACCGGATGCCACTGCCCGATTAATAGCGGCTACTTACTGCCTATCTTCAATGCCAATTATCCTGATAA

target 9: CCAAGAGAATGTGCTGTCTG - AGG (no off-target sequences with less than 5 mismatch)

Sequence used for searching

TTTGCCCTTCTTCCTTCCCCCCAAGAAATAAAAACGGGCACACATCAGGCATCAATTAAAATCCTCAGACAGCACATTCTCTTGGACGCTGGAGGTAATAGCCCGGTTAATAGCCTCATAATTAGCAATTCTTCGACAATTATTTTCCT

target 10: AAACCTGTATGTATCGGGAG - AGG (no off-target sequences with less than 5 mismatch)

Sequence used for searching

CTCTCCCTTGCCGCCGCGATGCATCTTGAAATCCATAGGGTATATACCATACAACCCTAAAGCCTCTCCCGATACATACAGGTTTGAAGCACACTTCATCACATCACTATTTTATTCACTCATCTATTTTTCTAAAAAAATCTATTTCC

Primers used for checking the 4 potential off-target sites for NRPS_7 (PluTT01m_16070) in

*Photorhabdus luminescens* subsp. *laumondi*i TT01 (NC_005126.1).

| Primer name | Primer sequence |
| --- | --- |
| Plumin-offtarget1.fwd | TGCCACTGTCGTCTCGAGTTGAG |
| Plumin-offtarget1.rev | TGGCGCAAGCGGTCATTCAATTC |
| Plumin-offtarget2.fwd | CGCCATTATCGTCTGAACTTAAGTGAAG |
| Plumin-offtarget2.rev | CGGCGCAGACGATCATTCAGTTC |
| Plumin-offtarget3.fwd | CGCCATTATCGTCTGAACTTAAATGAAG |
| Plumin-offtarget3.rev | CTGACGCAGACGGTCATTCAGTTC |
| Plumin-offtarget4.fwd | CTGACTACGCAAACGGTTATTCAGCTC |
| Plumin-offtarget4.rev | CGATATCGTATTGATCTGAAACGAGCAC |
