# Supplementary material for: Bacterial genome editing by coupling Cre-lox and CRISPR-Cas9 systems
Source: PLoS One. 2020 Nov 4;15(11):e0241867. doi: 10.1371/journal.pone.0241867 (PMC7641437; doi:10.1371/journal.pone.0241867)

Figure 2B_raw

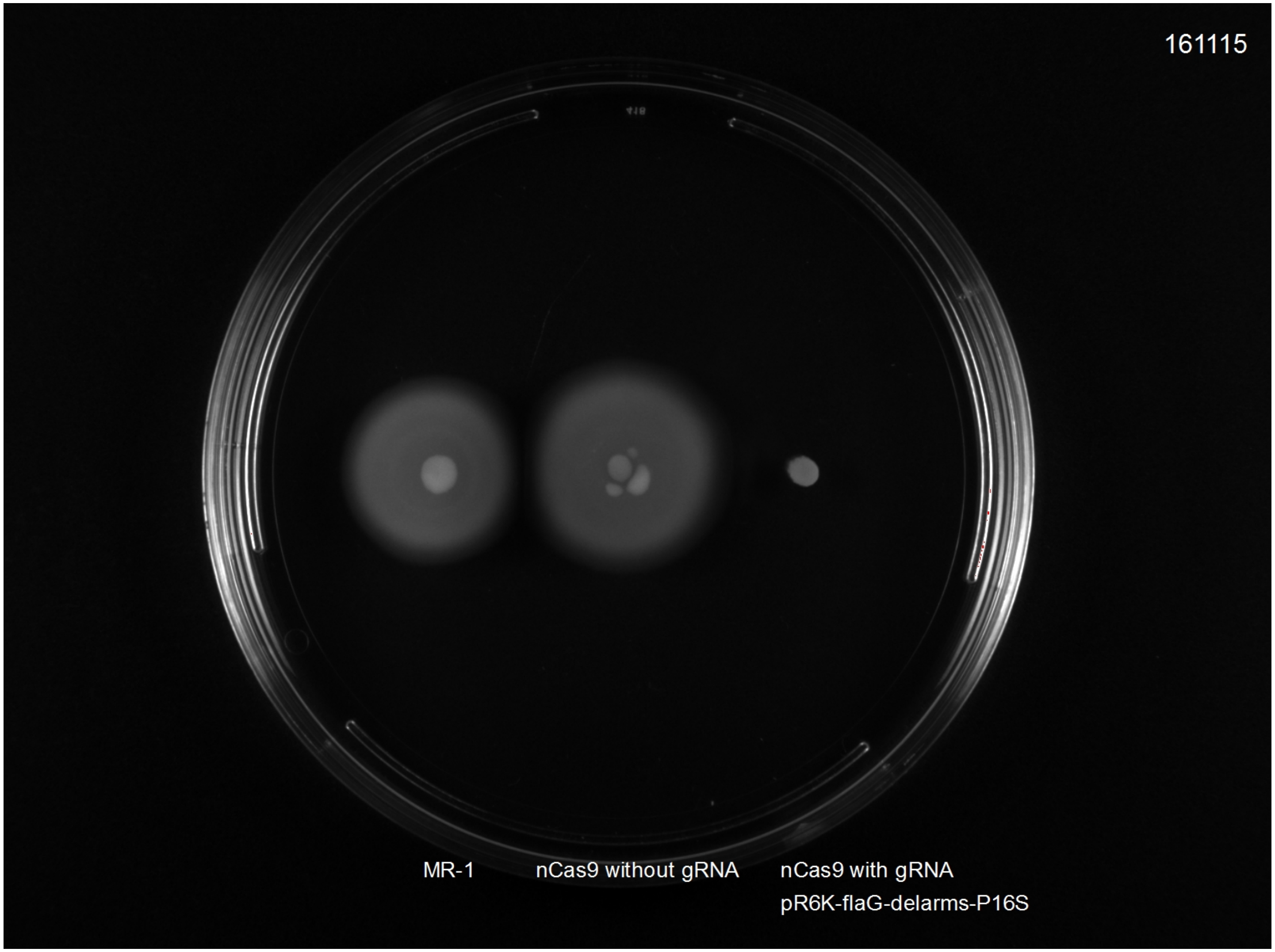

Figure 2C_raw

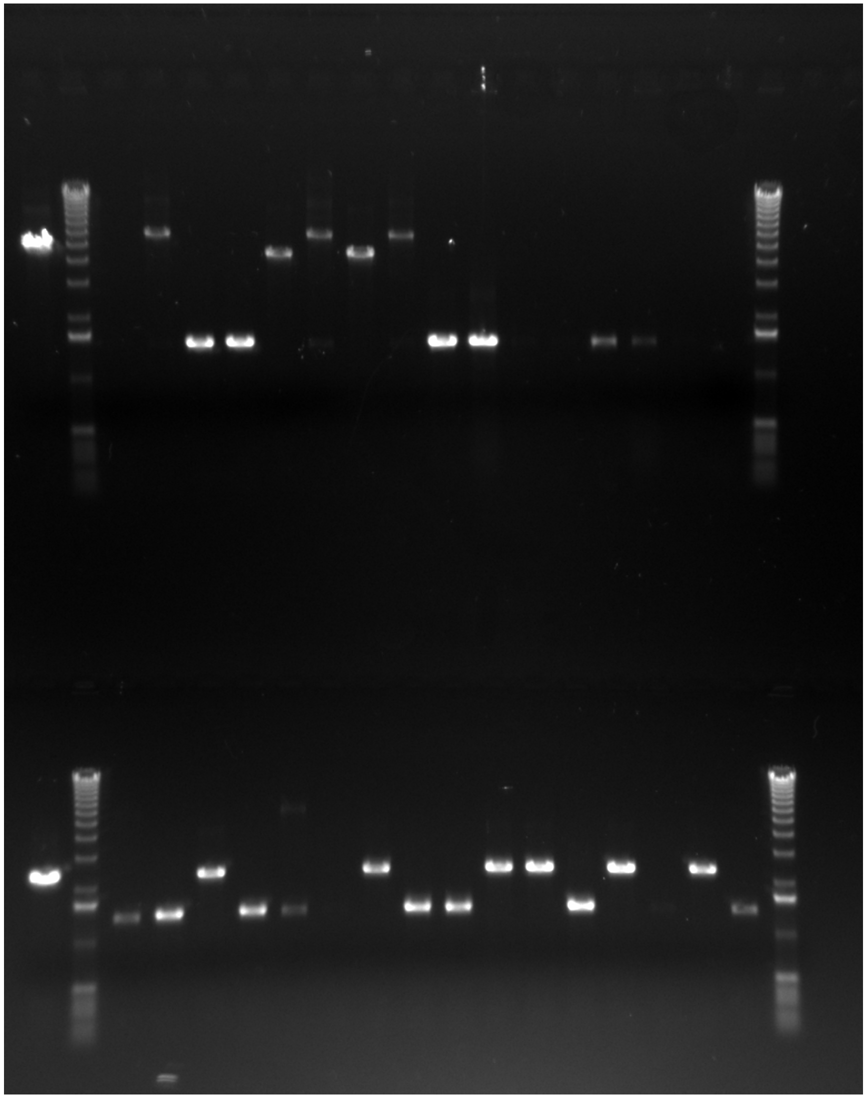

Figure 4C_raw

2019-12-17 photorhabdus LP cure screen and streak for paper

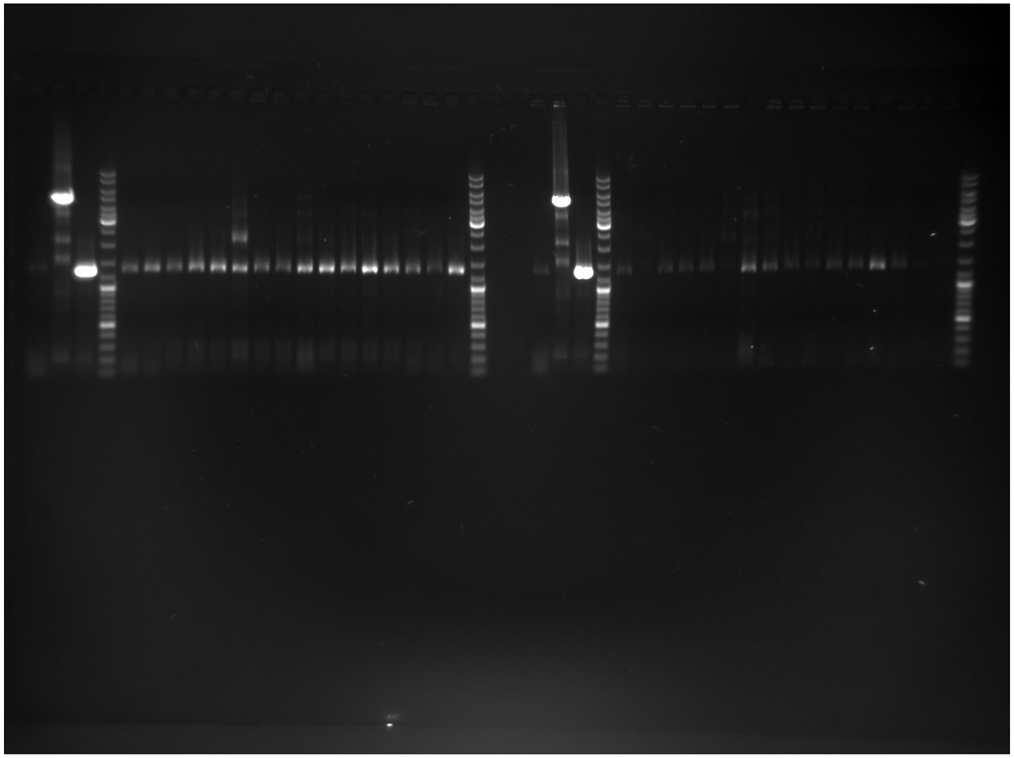

Location: //octopus/GenTech/Synthetic Biology/Dave/gel images BioRad
Printed: 12/17/2019 3:06:57 PM

Figure S3B_raw

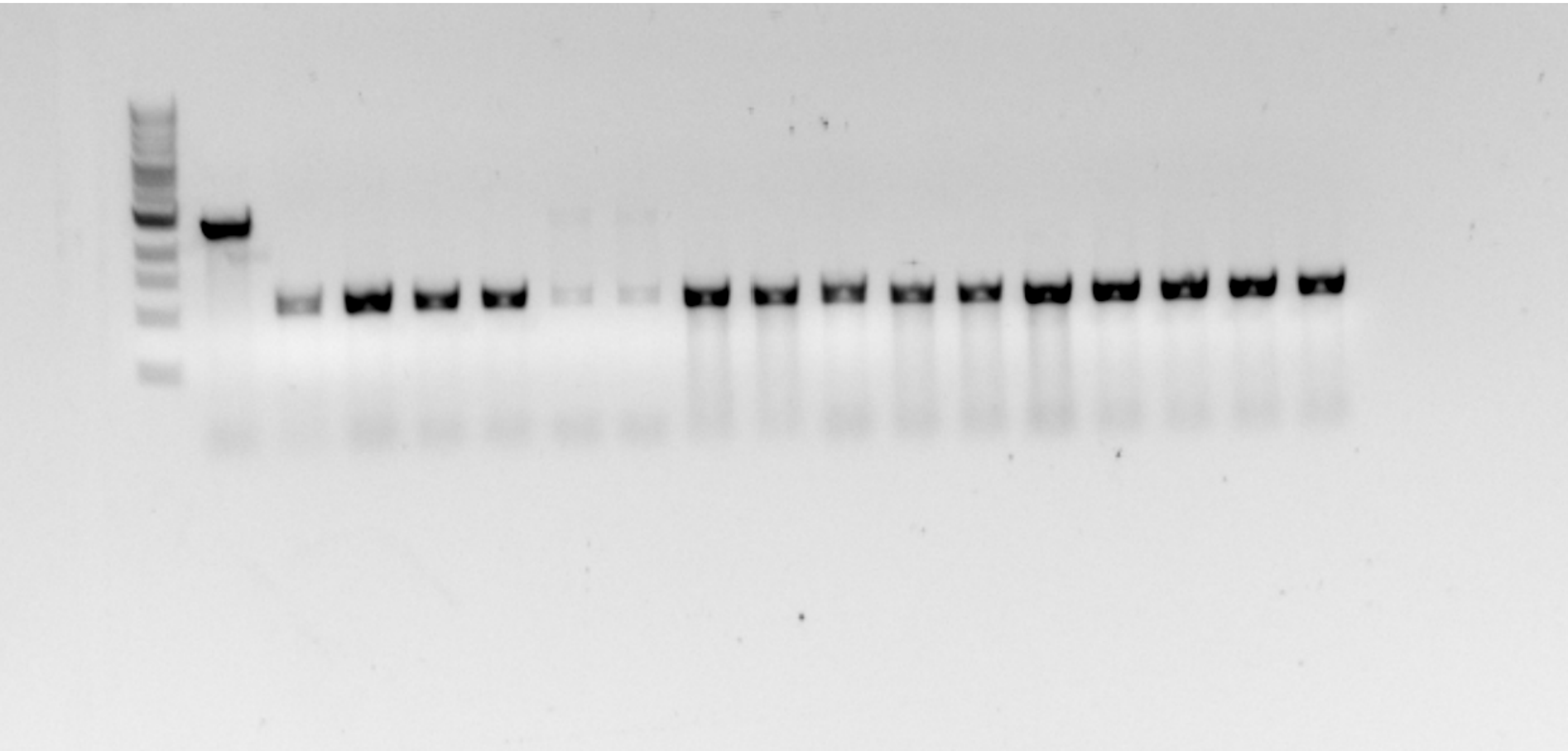

Figure S4B_C_raw

2019-12-16 Pseudomonas pvdI del screen and streak screen

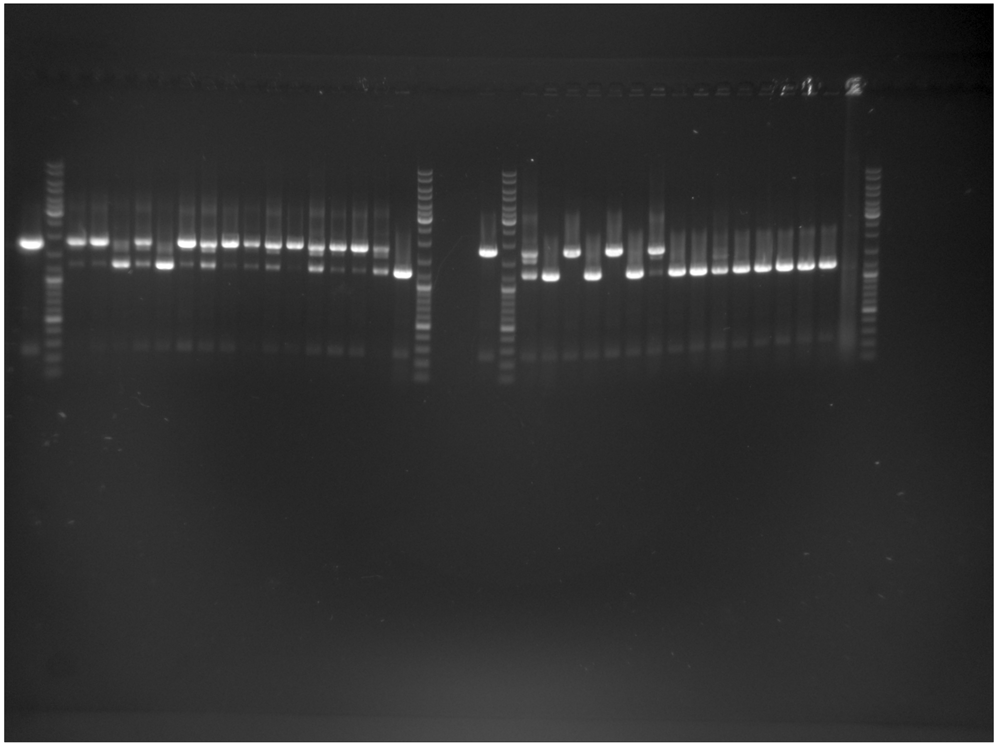

Location: //octopus/GenTech/Synthetic Biology/Dave/gel images BioRad
Printed: 12/16/2019 3:51:00 PM

Figure S5_NRPS_1_raw

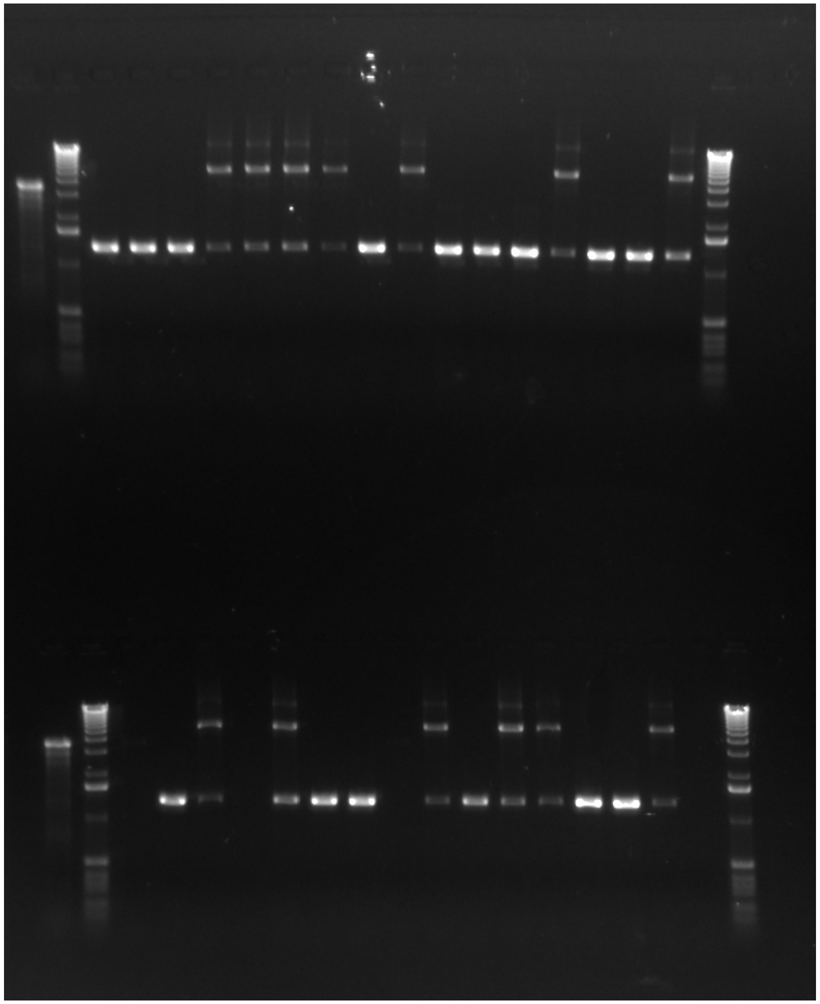

Figure S5_NRPS_2_raw

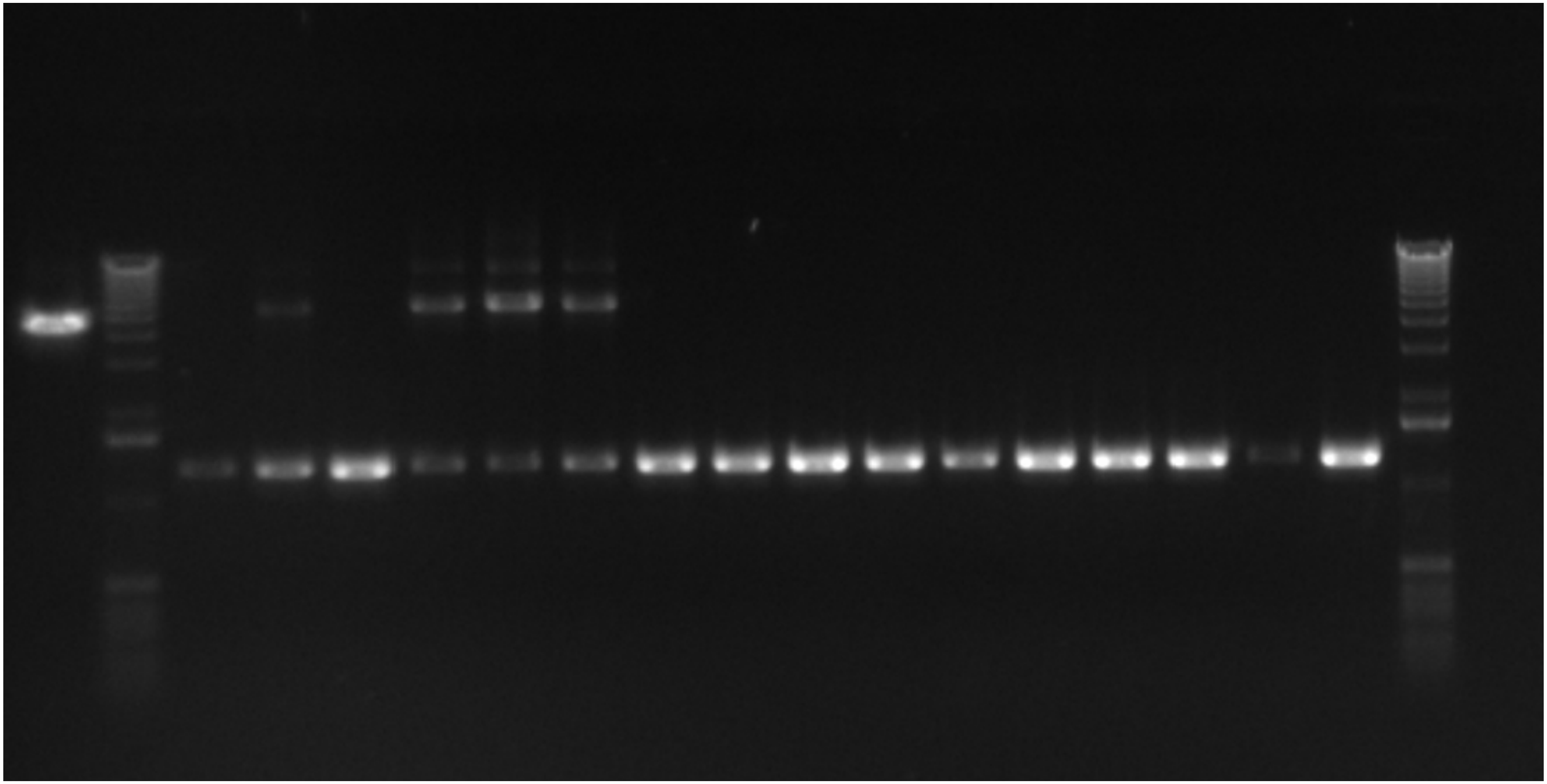

Figure S5_NRPS_3_raw_left

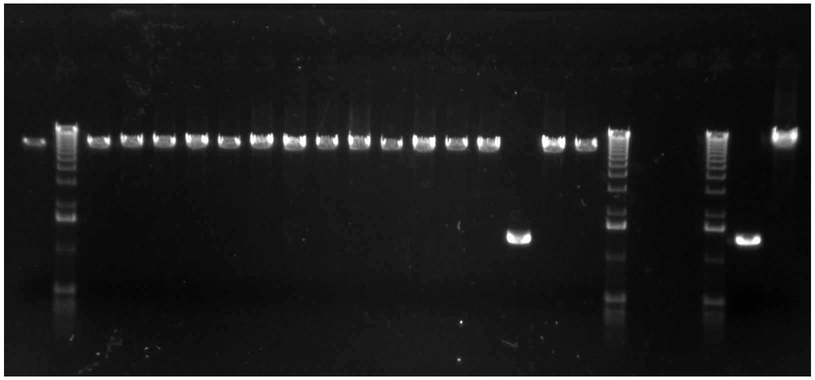

Figure S5_NRPS_3_raw_middle

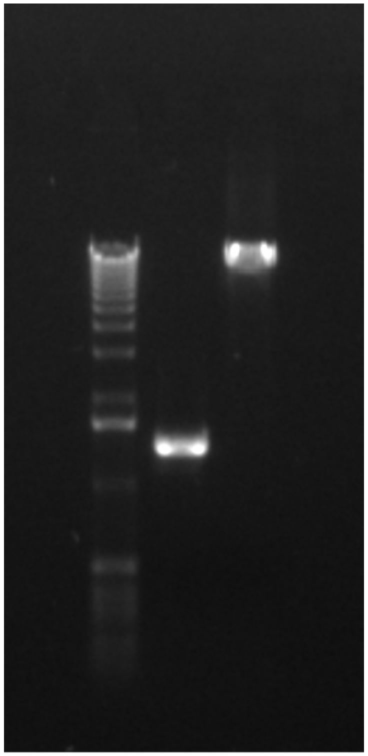

Figure S5_NRPS_3_raw_right

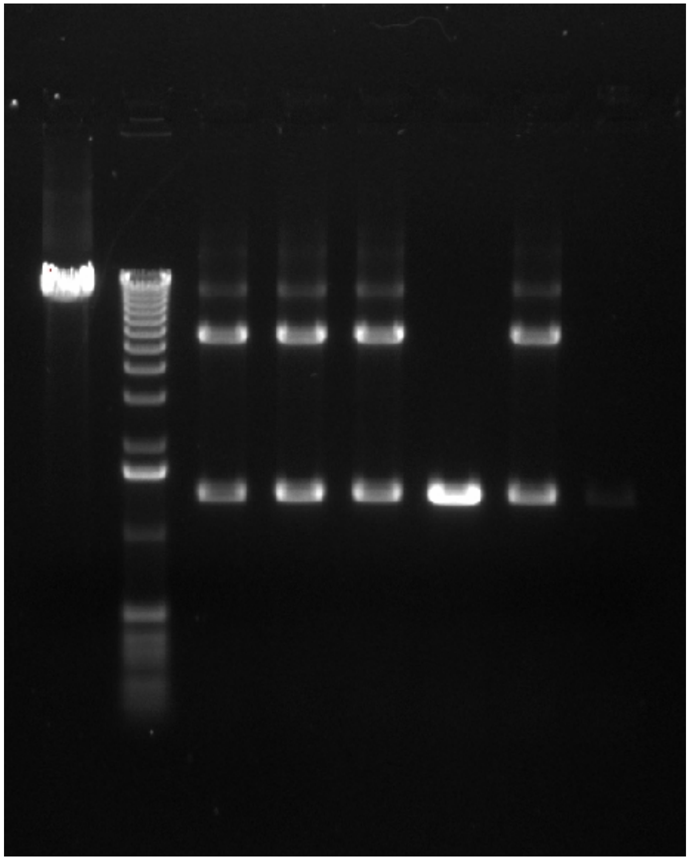

Figure S5_NRPS_4_raw

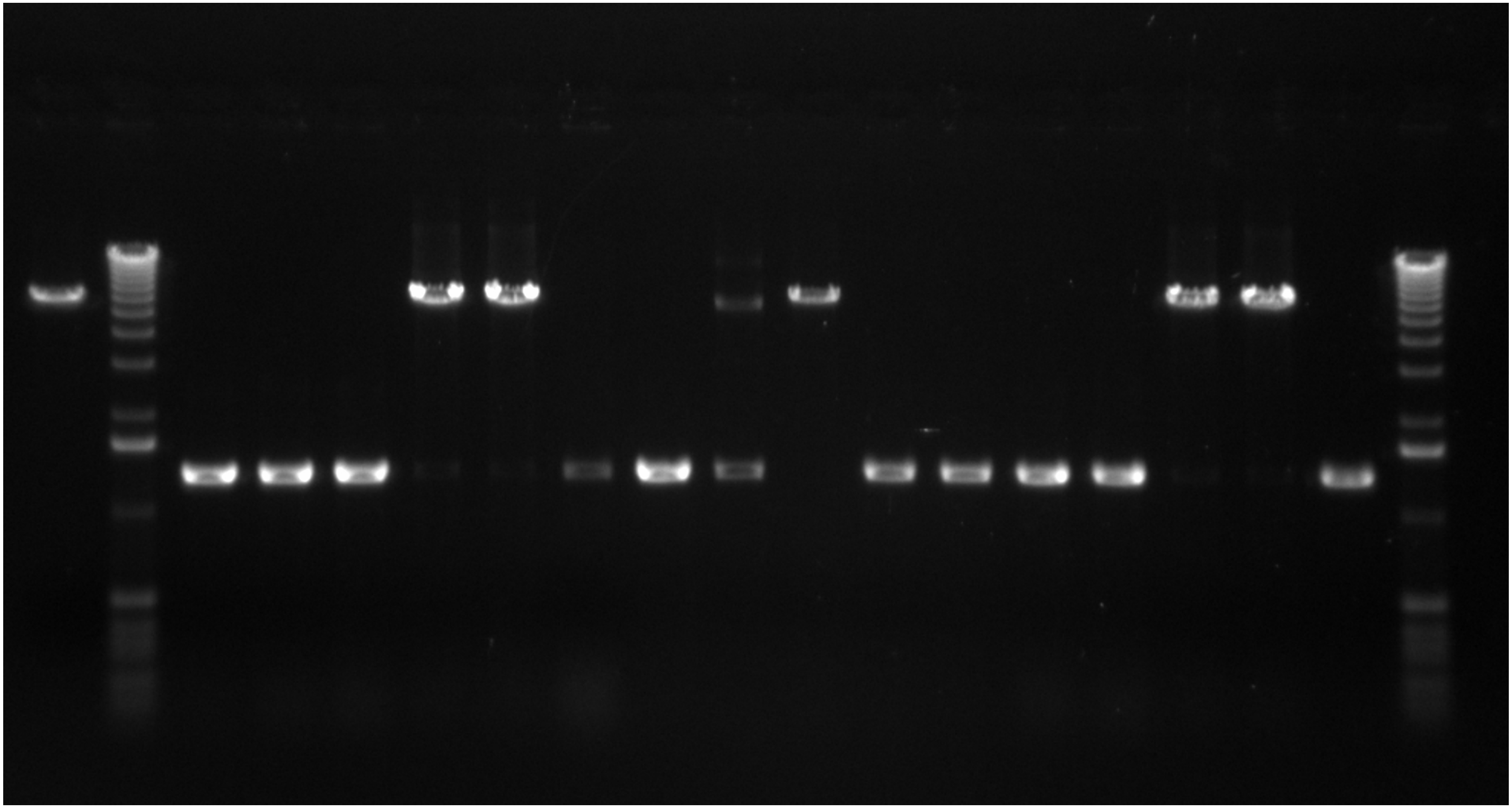

Figure S5_NRPS_5_raw

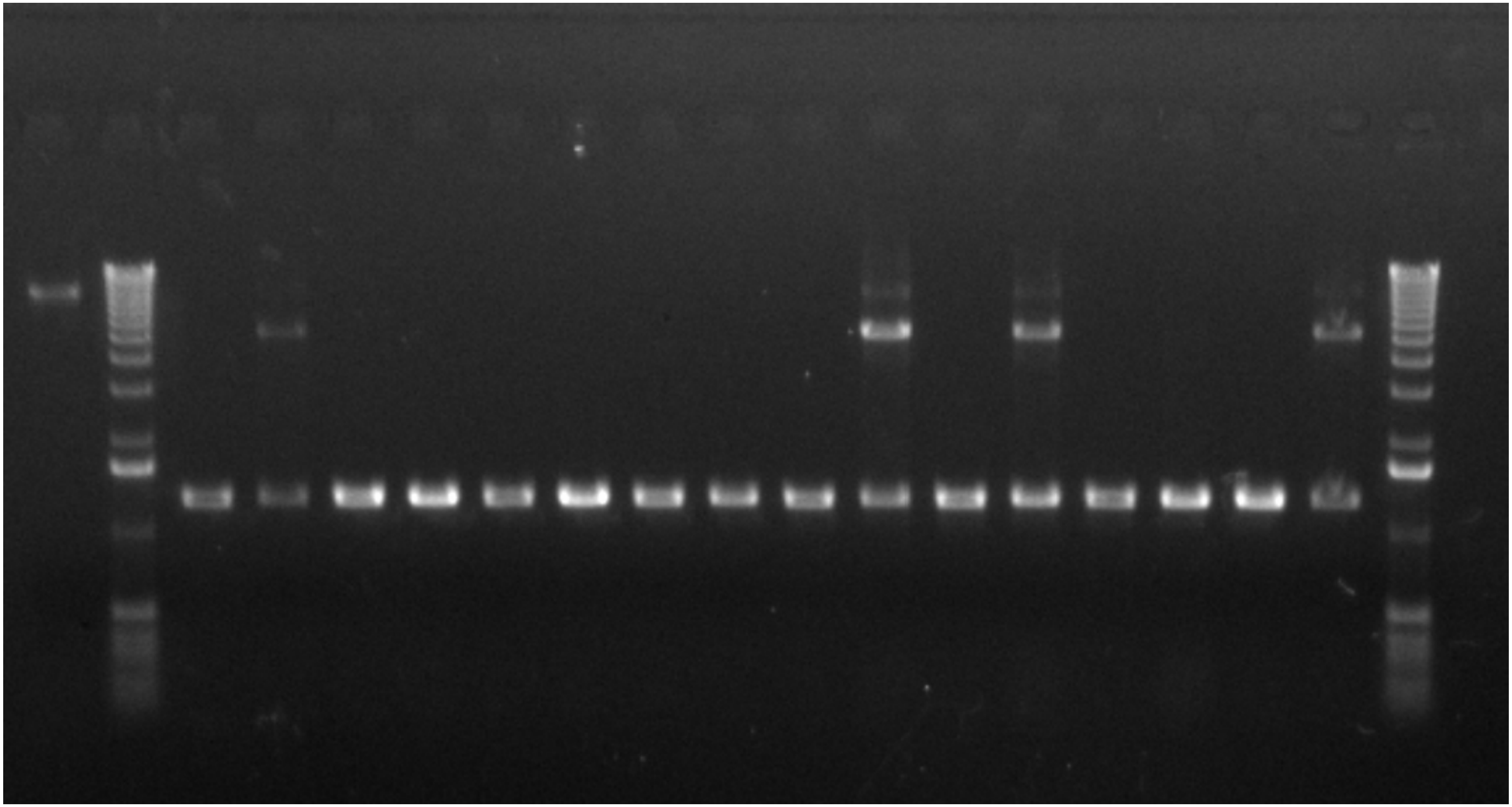

Figure S5_NRPS_6_raw

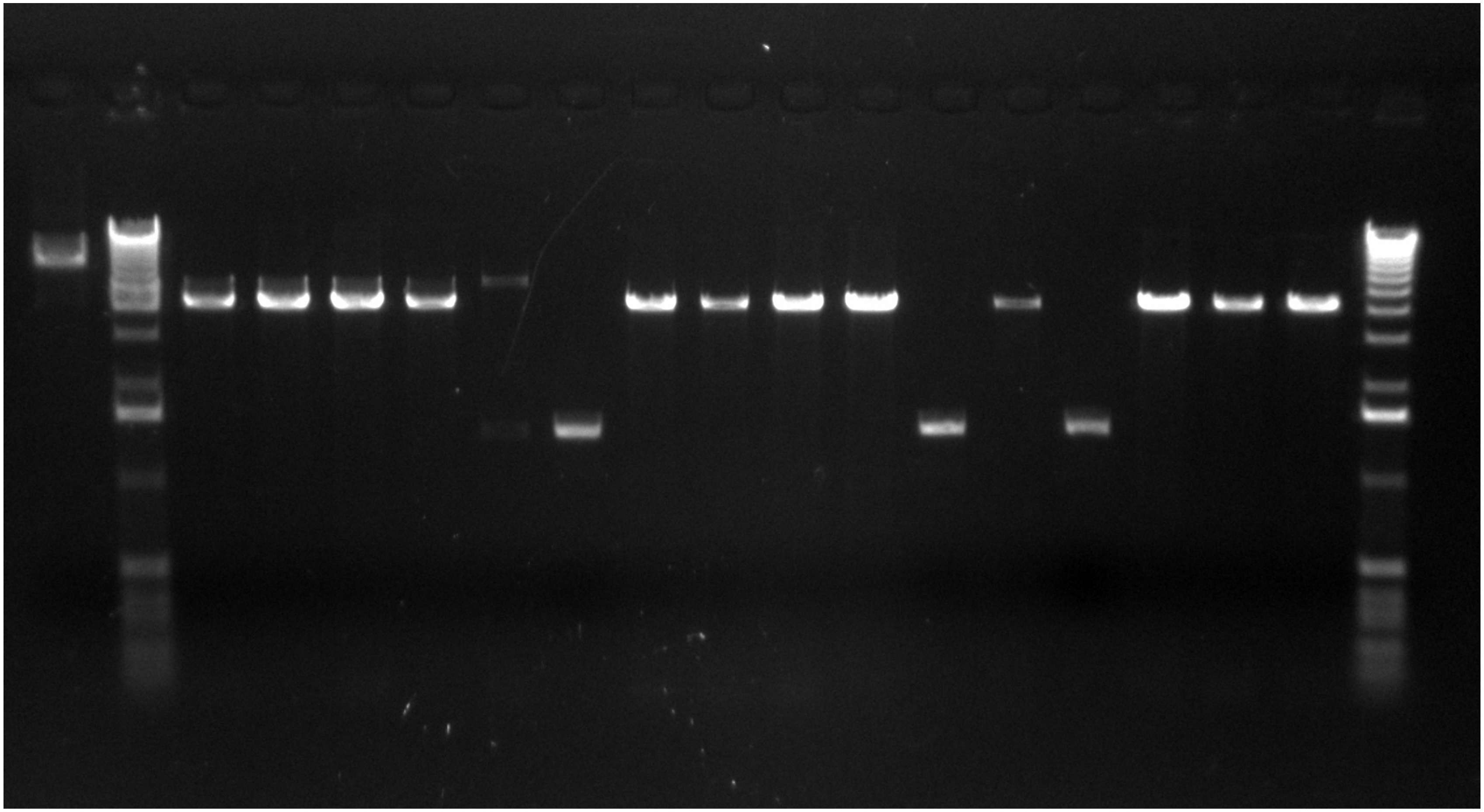

Figure S5_NRPS_7_raw

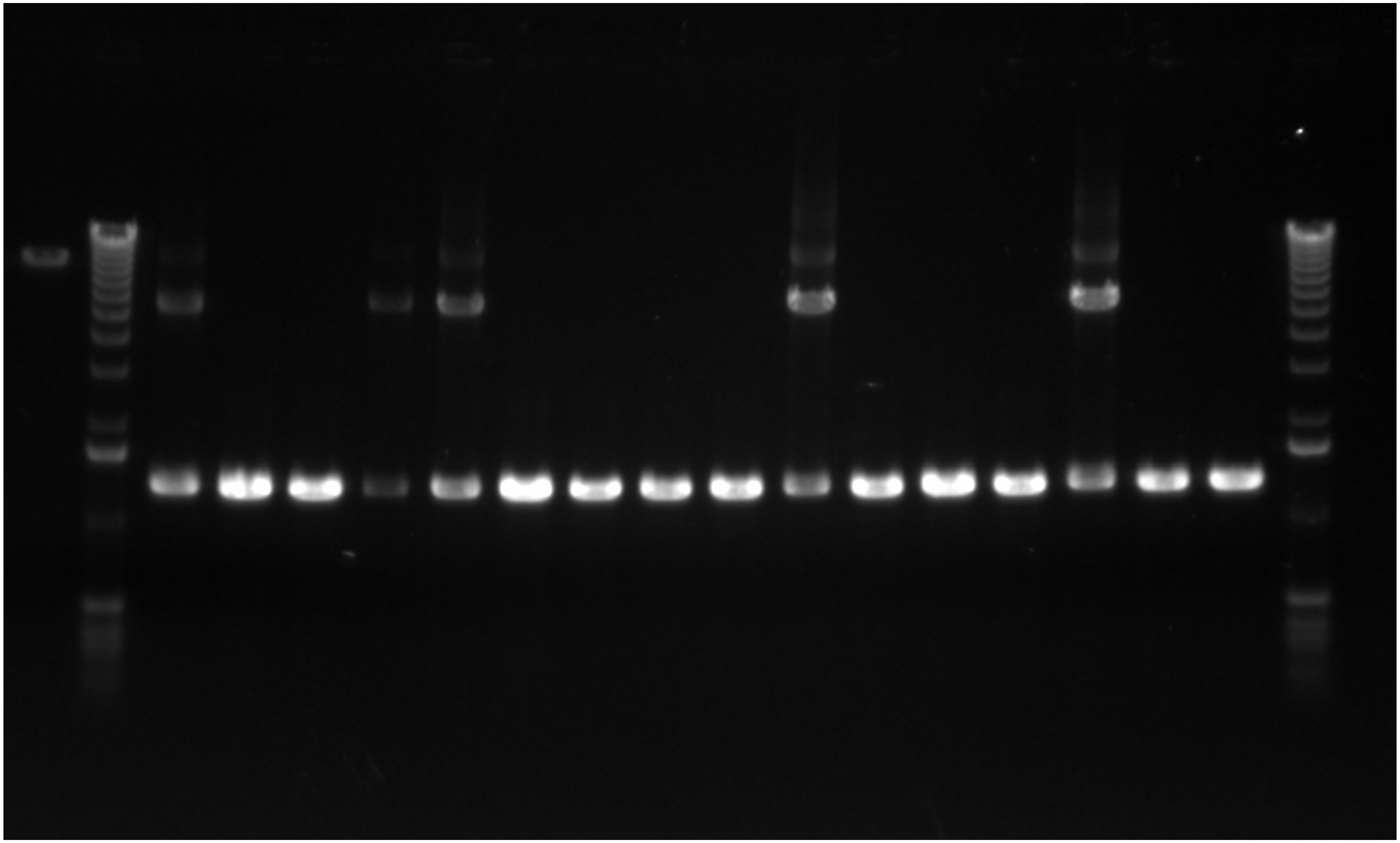

Figure S5_NRPS_9_raw

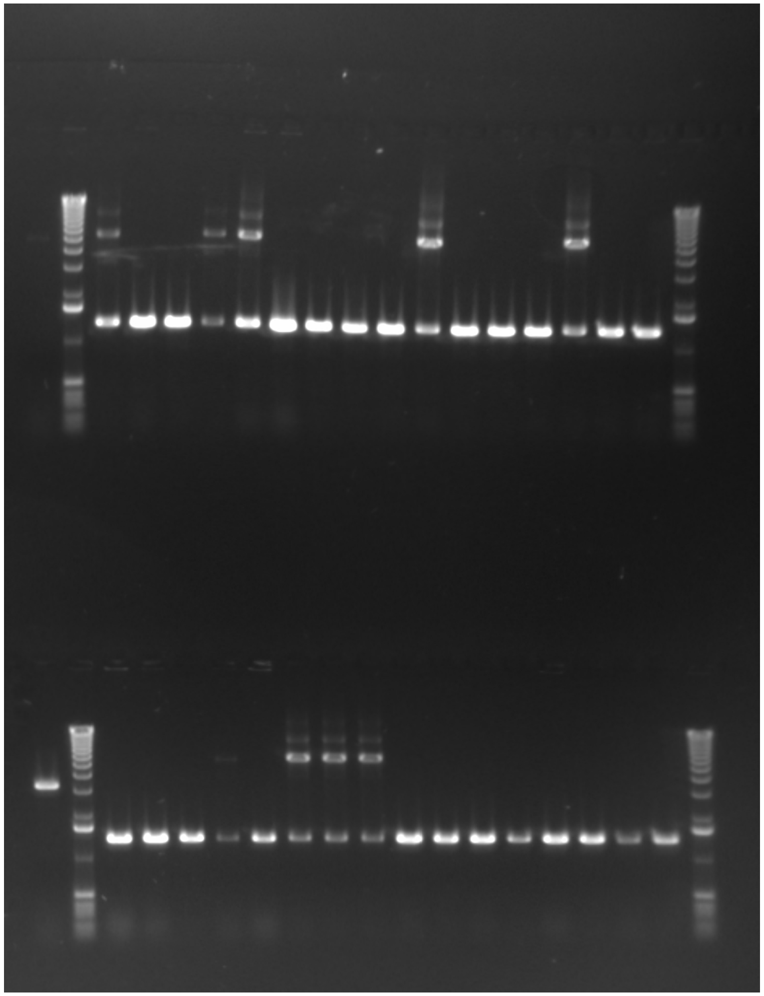

Figure S5_NRPS_10_raw

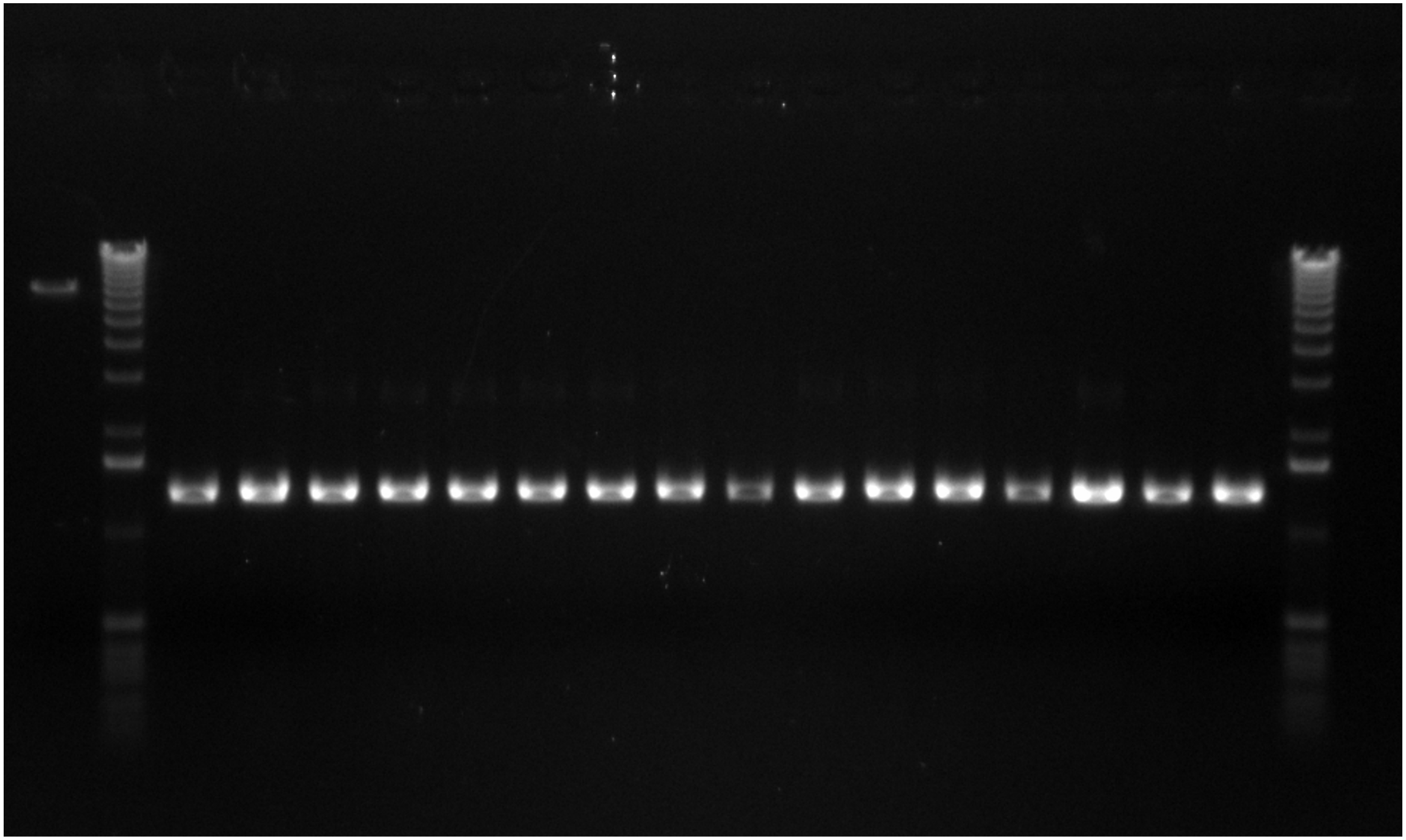

Figure S5_NRPS_11_raw

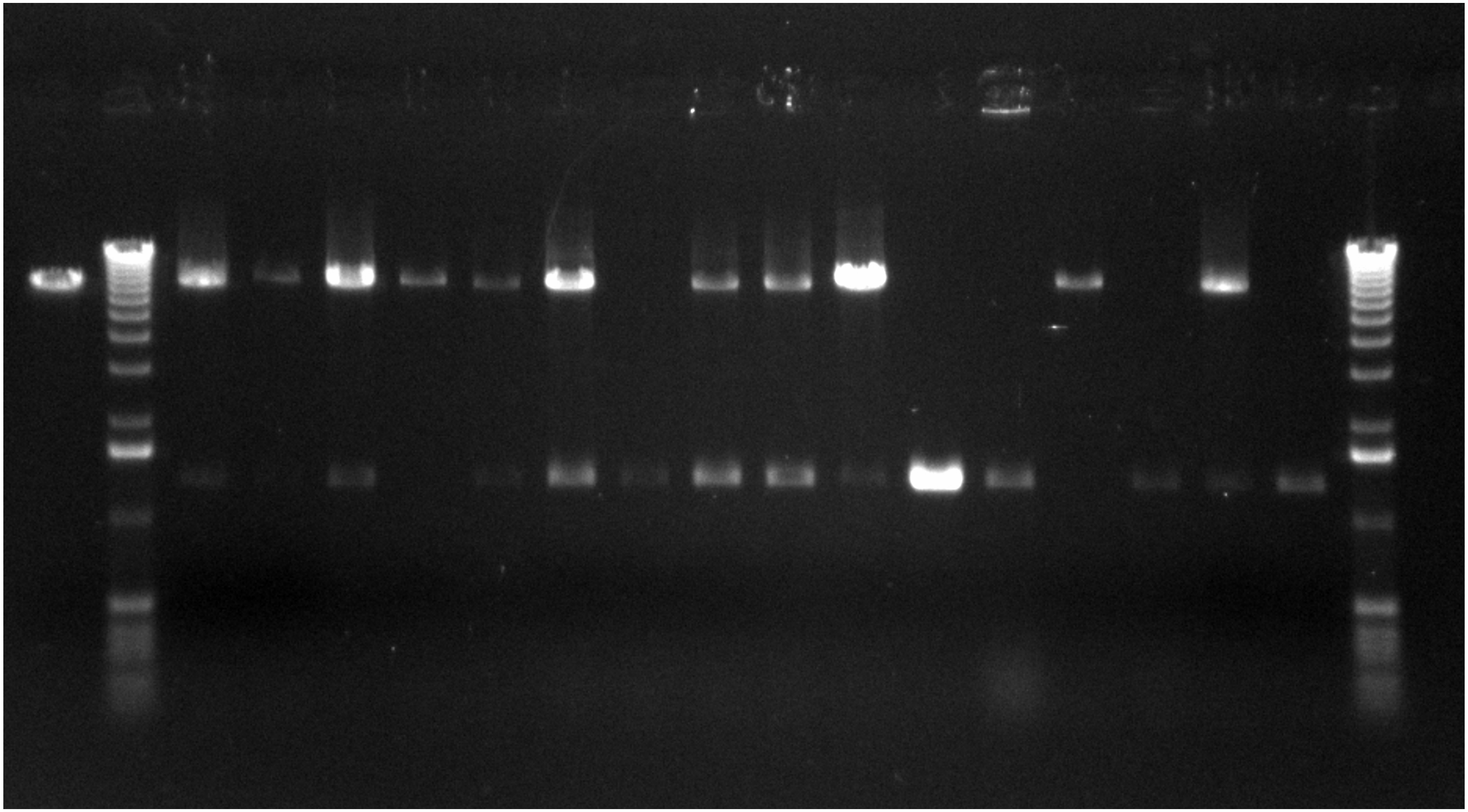

Supplement: S1 Raw images — (PDF) [file pone.0241867.s015.pdf]
